# Scenarios for the Altamira cave CO_2_ concentration from 1950 to 2100

**DOI:** 10.1038/s41598-024-60149-9

**Published:** 2024-05-06

**Authors:** Marina Sáez, David Benavente, Soledad Cuezva, Mireille Huc, Ángel Fernández-Cortés, Arnaud Mialon, Yann Kerr, Sergio Sánchez-Moral, Sylvain Mangiarotti

**Affiliations:** 1 Université de Toulouse CESBIO (CNES, CNRS, INRAE, IRD, UPS), Toulouse, France; 2 University of Alicante, Alicante, Spain; 3 Spanish Geological Survey, IGME-CSIC, Madrid, Spain; 4 University of Almería, Almería, Spain; 5 National Museum of Natural Sciences, MNCN-CSIC, Madrid, Spain

**Keywords:** Atmospheric dynamics, Climate-change impacts, Projection and prediction

## Abstract

A data-driven approach insensitive to the initial conditions was developed to extract governing equations for the concentration of CO_2_ in the Altamira cave (Spain) and its two main drivers: the outside temperature and the soil moisture. This model was then reformulated in order to use satellite observations and meteorological predictions, as a forcing. The concentration of CO_2_ inside the cave was then investigated from 1950 to 2100 under various scenarios. It is found that extreme levels of CO_2_ were reached during the period 1950–1972 due to the massive affluence of visitors. It is demonstrated that it is possible to monitor the CO_2_ in the cave in real time using satellite information as an external forcing. For the future, it is shown that the maximum values of CO_2_ will exceed the levels reached during the 1980s and the 1990s when the CO_2_ introduced by the touristic visits, although intentionally reduced, still enhanced considerably the micro corrosion of walls and pigments.

## Introduction

Underground atmospheres interchange greenhouse gases—such as CO_2_, CH_4_ and water vapour—with the outside atmosphere, following irregular seasonal patterns. The understanding of these exchanges is key to the preservation of valuable elements often found in caves. These include archaeological remains such as the parietal art of Altamira (Spain) or Lascaux (France), and unique biological and geological features such as speleothems, which can be used as paleoclimate records. Unfortunately, many caves are subject to considerable anthropogenic pressure because their stable environments can be easily disturbed by tourism or even by scholar activities, as a consequence of temperature changes, CO_2_ and water vapour exhalation, air currents, exogenous micro-organisms, etc. The study of cave micro-climates is also important since these exchanges may contribute to explain some imbalances found in the carbon cycle when only climatic and biotic contributions are considered^1–3^. Indeed, the reservoir of gaseous CO_2_ in caves could represent (if the effects of changes in land uses are excluded) up to 56% of the missing sink on an annual scale^4–6^. The knowledge of these exchanges is necessary to understand how the climate change will influence the accumulation or evacuation of greenhouse gases from caves to the atmosphere, and vice versa.

**Figure 1:**
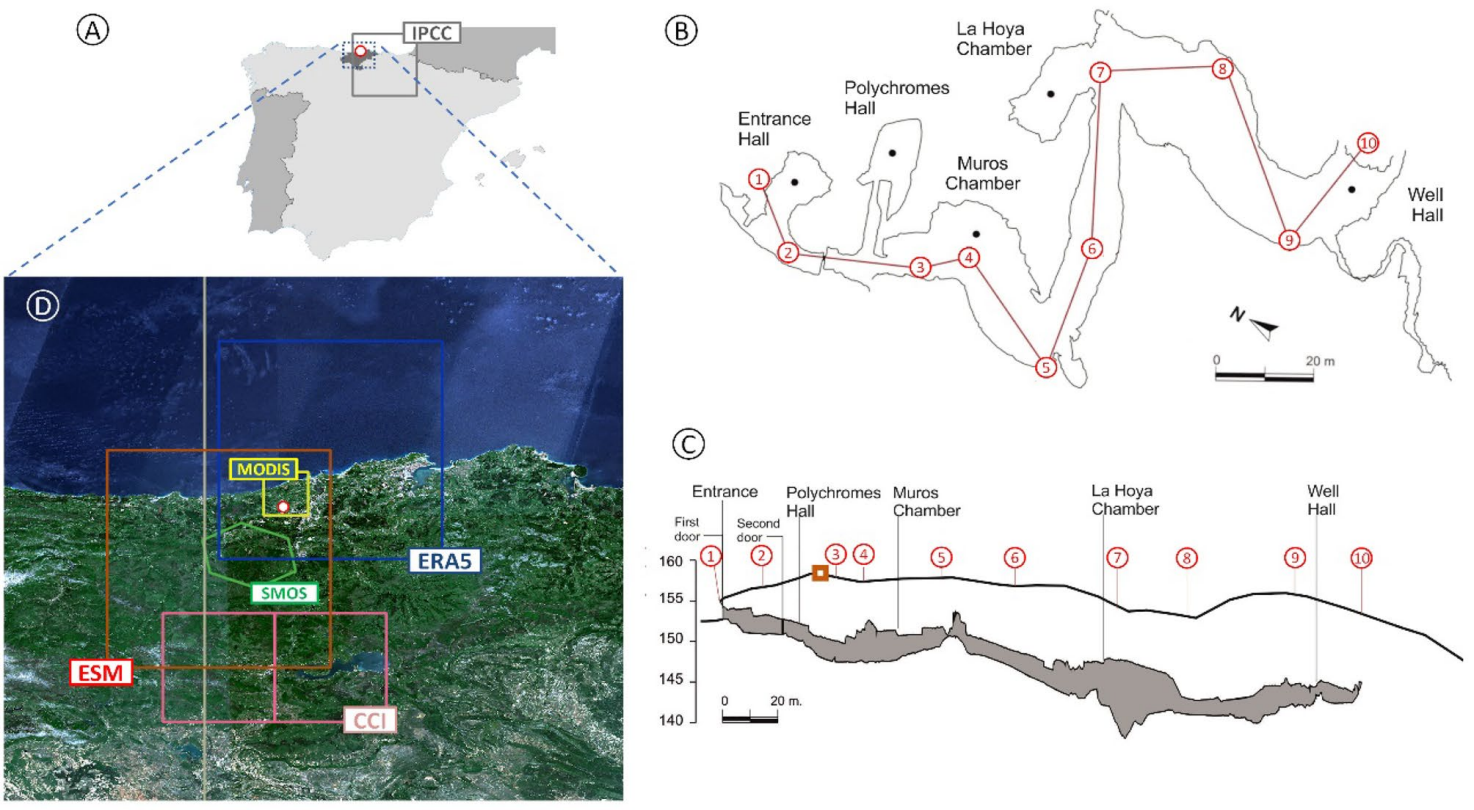
Area of study, data location and data footprint. Altamira is located North of Spain (43°22′40″N; 4°7′6″W) in the Cantabria province (**A**). It is a sloping down cave (**C**) with a single entrance located in the upper part of a 158 m high hill. The Polychromes hall is located close to this entrance in a connected hall located on the left after entering the cave (**B**). The concentrations of CO_2_ used in the study were all measured in this hall. The location of the in situ measurements performed outside the cave are also reported on (**C**) and represented by an orange square. The footprints (or grid pixels) of the other data sets used in the study are presented in (D): SMOS (in green), CCI (in pink) and ESM soil moisture (in red) for soil moisture, MODIS (in yellow) for land surface temperature, and ERA5 (in blue) for both soil moisture and temperature. The grid pixel of the IPCC model, too large to be plotted with the other footprints, is presented in (**A**). The maps were created with QGIS version 3.10 https://www.qgis.org.

The main objective of this study is to reconstruct the time evolution of the micro-atmosphere of the Altamira cave, located in Cantabria, North of Spain (see Fig. 1 and Supplementary Note 1 for details), and, more specifically, its variations of CO_2_ concentration in the long term. Previous studies have focused mainly on the statistical relationships between the factors at play in the cave, leading to a good overview of all the processes at work. In particular, the functional relationships between in-cave and external micro-climatic parameters and the variations of trace gases have been investigated based on the concept of entropy^7,8^. However, the dynamical links between the variables remain poorly known: the governing equations are not available. It is therefore difficult to perform simulations and to generate scenarios of both the past and the future. Although challenging, such projections are necessary to anticipate its coming evolutions. In order to make progress in this area, this study presents two dynamical models for the CO_2_ concentration in the atmosphere of the Altamira cave, reconstructed by a data-driven approach. In a previous research, it was found that the cave micro-climate exhibits low-dimensional chaotic behaviour. This result was supported by three dynamical models based on the radon concentration^9^. In this work, we focus on the CO_2_ concentration in the Altamira cave and on the two main drivers for the cave’s micro-climate: the outside temperature and the soil water content^10^.

The study of the Altamira underground micro-climate is key to the preservation of its Palaeolithic paintings, threatened by decades of intense touristic exploitation and anthropogenic modifications in the cave. It has proven to be particularly sensitive to the increase of CO_2_ concentration and temperature, which imbalances the air exchange pattern with the exterior and, eventually, fosters the entry and dispersion of outdoor nutrients and micro-organisms linked to bio-deterioration processes of rock-paintings^11,12^. Caves follow seasonal ventilation patterns, which are triggered by the density difference between the inner and the outside air. For caves located in non-tropical latitudes, the most important factor controlling these differences is the temperature^13,14^. In Altamira, between the end of October and mid-November, the CO_2_ coming from the upper soil accumulates in the cave due to lack of ventilation (see Supplementary Fig. 1). By mid-May or mid-June, the air of the atmosphere becomes hotter than that of the cave, allowing ventilation and, consequently, the concentration of CO_2_ in the cave decreases^15^. Although this behaviour contrasts with the classical models for cave ventilation through openings^16,17^, it has been well established for the Altamira cave in previous studies^10,18^. The annual cycle of charge and discharge is apparent, although partly blurred by episodes of short time scale^10,19^. The volume of water contained in the soil layer, on the surface above the cave, has been proposed as another important factor controlling these exchanges^20,21^, since the Altamira cave is located in a humid area with moderate evapotranspiration. In the first place, the soil above the cave is the main source of CO_2_, which is transported by diffusion to the cave atmosphere^10^. In addition, the volume of water in the soil can enable or disable the passage of gases^22^. A dynamical coupling between the volume of water in the soil and the gas concentration in the cave atmosphere (^222^Rn) has been be evidenced^9^. This coupling suggests that a lower content of water promotes out-gassing and allows faster gas interchange and vice versa. Nevertheless, no model able to account for the CO_2_ and its main climatic drivers could be proposed and the past and future evolution of the cave micro-atmosphere remains an important problem to investigate.

When choosing an approach to tackle this problem, four main criteria should be considered:

- (1) A data-driven approach should be preferred as no model is currently available, and because the data presently available would not suffice to constrain any process-based model satisfyingly;
- (2) The chosen approach should be, in its principle, independent of the initial conditions because the problem presents a sensitivity to these initial conditions caused by the climatic forcing;
- (3) It should not lead to an inaccessible black box: instead, ideally, it should offer a reusable formalism, enabling to deepen the problem further and to investigate new hypotheses;
- (4) It should be applicable to relatively small data sets, since in practice, only few cycles of observations are generally available in cave research; this rules out methods such as artificial networks or deep learning;
- (5) It should be sufficiently robust to be applied to environmental datasets from the real world.

Initiated at the beginning of the 1990s, the global modelling technique used in the present study can be an efficient alternative to more common approaches. It is based on chaos theory and is, therefore, well adapted to deal with unstable dynamics, which must be considered as a very plausible hypothesis as far as atmospheres are concerned, as illustrated by the theoretical system introduced by Edward N. Lorenz in the early 1960s^23^.

Chaotic dynamics pose difficult problems for scientists^24,25^, basically because slight differences in the initial conditions can give rise to very different time evolutions. A modelling approach well adapted to these types of dynamical regimes must be able to retrieve the original dynamics whatever the original conditions and their time evolutions are (that is, from any time evolutions resulting from the same governing equations, whether they start from very similar or very different initial conditions).

The global modelling technique^26^ was especially designed for this purpose. It assumes that the governing equations of the studied systems are of deterministic form and can thus be written as

1 $${\text{d}}{\mathbf{X}}/{\text{dt }} = {\mathbf{\mathcal{M}}}\left( {\mathbf{X}} \right),$$

with **X** a state vector of the effective variables of the studied system and **ℳ** its governing equations. Its aim is to unveil these equations based on a set of observed variables. Initiated in the early 1990s, the approach was explored both from univariate^27–30^ and multivariate time series^28,31–33^.

In this latter case, the global modelling technique may become a very interesting tool to extract information in an algebraic form, directly from observational time series, making the black box transparent, as far as possible. Indeed, the approach was proven very powerful to retrieve the exact structure of the original equations when these are of compact form (i.e. equations that cannot be simplified further or the original dynamics will be lost), or to obtain a proper approximation of them if not, even under degraded measurement conditions^33^. The approach can also be used to detect weak directional couplings from small subsystems involved in a higher dimensional network. As a general technique, of course, it can also be applied to the particular case of non chaotic behaviours.

The global modelling technique could be applied to experimental problems since the mid 1990s^26,34^. It is more recently that it was applied to environmental data from the real world^35^, for instance in hydrology or soil eco-hydrology. In a case of eco-epidemiological application, for the outbreak of bubonic plague in Bombay (1896–1911), the approach even proved to be able to get interpretable equations directly from observational time series^36^. Closer to the problem that interests us here, it was applied to the atmosphere of the Altamira cave. Two bivariate chaotic models were obtained for the dynamics of CO_2_ and ^222^Rn, suggesting a regime close to chaos^9^.

This technique requires very few assumptions. It implicitly assumes that the phenomenon is deterministic. In the present study, it is also assumed that the dynamics is either polynomial or approachable by means of polynomial equations. Other than this, the global modelling technique does not require any assumptions about the physical processes involved, neither the calibration of empirical or semi-empirical coefficients.

In addition, this technique allows modelling even when some variables involved in the dynamics were not observed, which is a very common situation in real conditions, especially when dealing with the underground. In other words, the original dynamics (with all the active variables) may be reformulated based on some of the observed ones. In order to account for the missing variables, the models include derivatives of the observed ones, which is grounded in the embedding theorems^37–39^. In practice, it is not always possible to replace the active variables in a reformulation of the dynamics involving derivatives of the observed variables. This is a question of observability^40^, which cannot be anticipated. However having found a global model for a phenomenon implies that it was indeed observable.

All these strong properties (insensitivity to the initial conditions, robustness to measurement and dynamical noises, as well as to subsampling and gaps, ability to work from a restricted number of variables, possibility to get algebraic formulations and potentially interpretable ones, and capacity to tackle with observations from the real world) have made the global modelling a well designed tool to work on the micro-atmosphere of the Altamira cave. Moreover, all these advantages make it possible to work on problems whose dynamical equations are unknown or poorly known, and poorly constrained as well, due to the limited accessibility of the underground. It also makes possible to work on problems that involve multidisciplinary realms such as physics, biology, geology, hydrology for which no coupling models generally exist.

### Chaotic models for Altamira dynamics and its drivers

In this research, univariate analyses were first applied to the variable *C* the concentration of CO_2_ inside the cave, and to two external variables identified as drivers of the dynamics of the micro-atmosphere: (i) *T* the temperature of the outside atmosphere, which is at the source of the density gradient with the micro-atmosphere air^41^; and (ii) *W* the Volume Water Content (VWC) at the vertical of the cave, assumed to play an important role by fostering/hampering the exchanges through the soil^9,20,22^. The Altamira cave is located in the upper vadose zone of a senile karstic system and it is hydrologically unconnected, therefore no significant aspects of the groundwater in the unsaturated zone need to be considered in the model.

Details about the data, their preprocessing and their intercalibration are provided in Supplementary Note 2 (see also Supplementary Table 1, 2, 3). A general description of the used algorithm is shown in Supplementary Fig. 2 with all details, including the validation procedure, provided in the Methods section.

**Figure 2:**
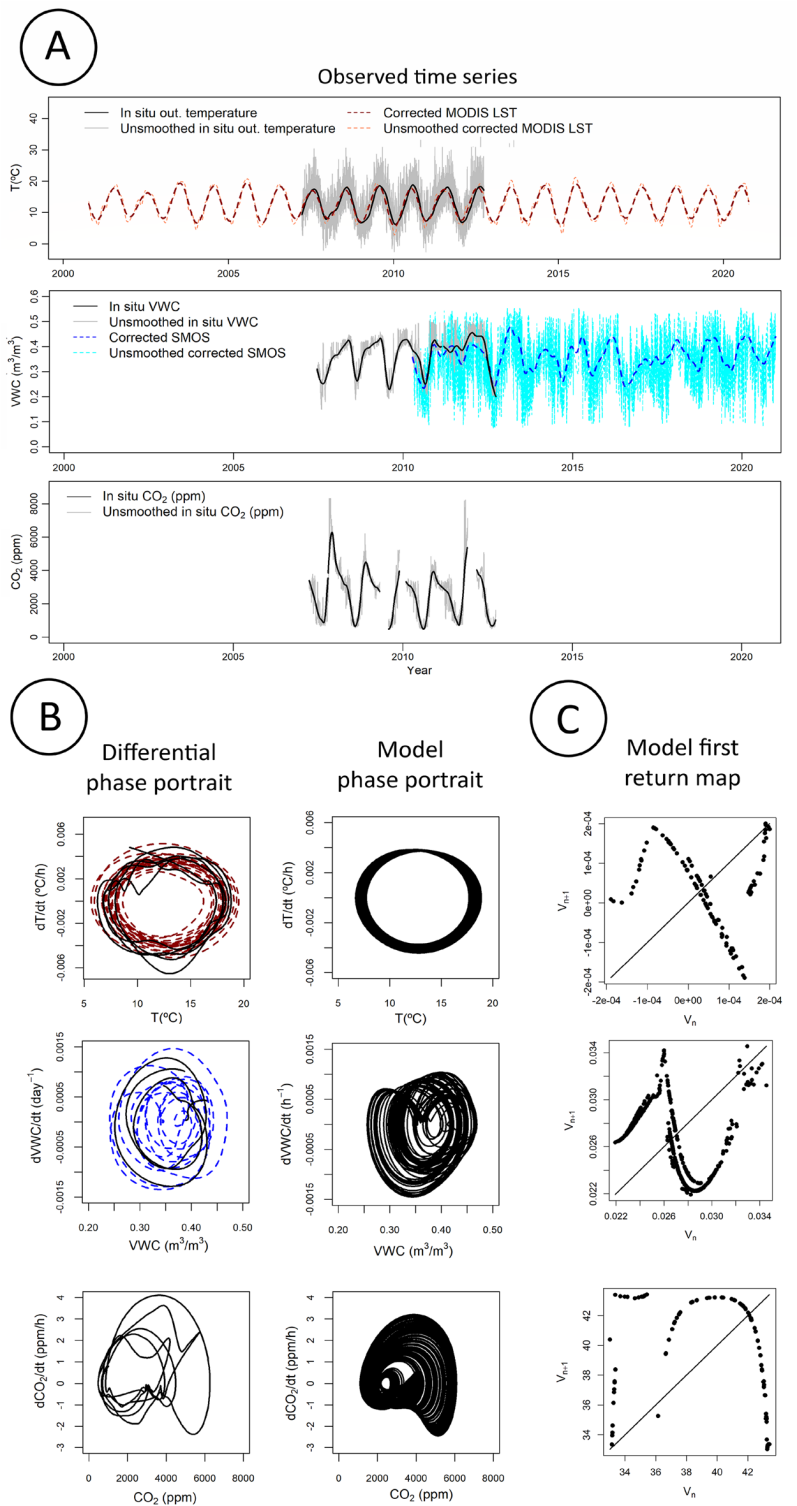
Univariate modelling. Three observed variables were considered in the study, which time series are shown in (**A**): the outside temperature *T*(*t*) measured in situ (shown before (grey line) and after (black line) smoothing) or estimated from MODIS satellite product (also shown before (orange dashed line) and after (red dashed line) smoothing), the volume water content *W*(*t*) measured in situ (shown before (grey line) and after (black line) filtering) or estimated from SMOS satellite (also shown before (cyan dashed line) and after (blue dashed line) filtering), and the concentration of CO_2_ *C*(*t*) measured in situ only and shown before (grey line) and after (black line) smoothing. (**B**) Differential phase portraits reconstructed from these smoothed time series with both the in situ (plain lines) and the satellite based (dashed lines) reconstructions. (**C**) The phase portraits and first return maps by applying the global modelling technique to these time series considered separately, these maps all exhibit multiple branches characteristic of chaotic dynamics.

The univariate models **U**_C,_ **U**_*T*_ and **U**w were obtained for each variable considered in this study (Fig. 2A–C, see Supplementary Note 3 for details). These models converge to chaotic attractors and they enable to gather in a single and consistent framework all the properties of chaos (summarized in Supplementary Table 4): determinism^42^, fractal dimension^43^, high sensitivity to the initial conditions^44^, folded structure^45^ of the attractor, and robustness to long term numerical integration^46^. The dynamics of the open atmosphere is highly sensitive to the initial conditions as expected theoretically since the pioneering works on chaos^23,24,47,48^, which is confirmed here—directly from observational time series—by obtaining chaotic attractors for the variables *T* and *W*.

**Figure 3:**
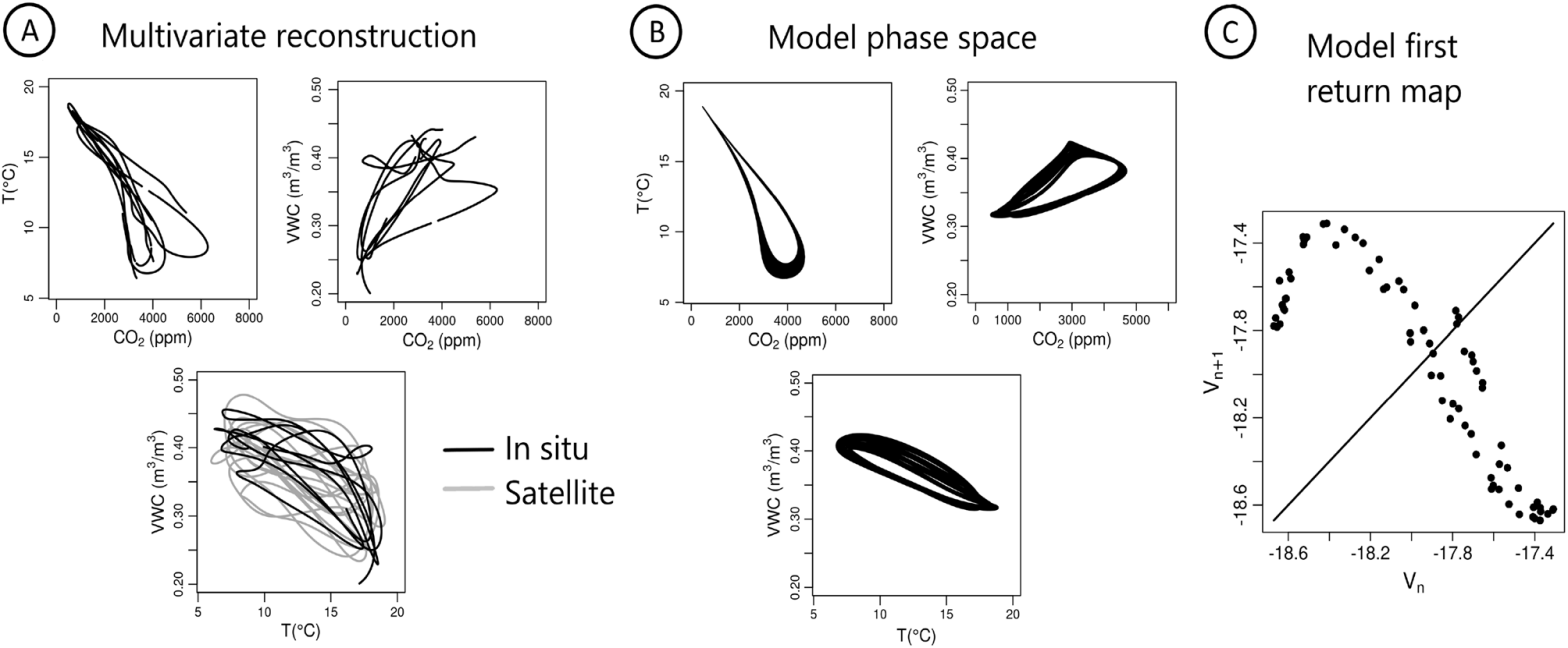
Multivariate modelling. Projections of the multivariate phase space reconstructed from the observational time series are shown in (**A**), based in situ data (all the projections) and satellite data (when available). The multivariate phase portraits of the model M_*CTW*_ obtained by applying the global modelling technique to the time series considered together is shown in (**B**), and its first return map in (**C**) characterized by two branches characteristic of chaos.

Since the cave is not isolated from the open atmosphere, the chaotic regime of the CO_2_ concentration may not result from internal mechanisms but rather from the coupling to external factors. To investigate this question, the global modelling technique was applied again, considering the three time series as input, with the purpose of unveiling underlying couplings between *C* the concentration of CO_2_ in the cave and the two expected external drivers *T* and *W*. Among the models not rejected by the procedure, a single model **M**_*CTW*_ was able to reproduce the dynamics of the three variables, and their coupling, as observed in Fig. 3. Details about the procedure used to obtain this model are provided in Supplementary Note 3. This model enables a rough but consistent reconstruction of the phase space and gives good forecasting performances in the short term. As a result of the removal of retroactions within the model structure, this multivariate model **M**_*CTW*_ highlights the independent dynamics of the outside variables *T* and *W*, which together play as unidirectional forcing on the concentration of CO_2_ in the cave. Moreover, it generates chaotic dynamics for the three variables *C*, *T* and *W* as expected, and the oscillations of the three variables all have pseudo-periods close to annual because *C* and *W* are synchronized on the annual pseudo-period generated by the sub-model **U**_*T*_ (Eqs. 16–18 in **M**_*CTW*_). It can be considered as a proper reconstruction of the original dynamics in terms of types of trajectories. The chaotic dynamics of the micro-atmosphere can be explained, therefore, by the chaotic dynamics of the two drivers.

In Supplementary Fig. 1, it is shown that in 2011 the in situ soil moisture cycle was greatly disturbed by a very rainy summer, however, the CO_2_ continued its annual dynamics following the temperature cycles. This fact is accurately reproduced by the model, where, as explained, the CO_2_ concentration always synchronizes with the temperature, which is, therefore, the main factor triggering the basic cycle of ventilation and accumulation. The basic shape of an annual CO_2_ cycle consists of: (i) a rapid recharge; (ii) a slight out-gassing taking place when the air is still trapped in the cave (the CO_2_ probably moves by diffusion to lower, CO_2_-depleted parts of the karst system); (iii) a stabilization around 3000 ppm; (iv) a rapid discharge, when ventilation is triggered by the temperature; and (v) a stabilization close to the CO_2_ concentration of the outside atmosphere. This basic shape results from typical temperature and the CO_2_ cycles. However, the soil moisture time series tends to show irregular cycles and acts often as a perturbing factor. The soil moisture content, therefore, enhances the complexity of the CO_2_ behaviour, resulting mainly in higher or lower maxima and in orbits with various shapes. This confirms the results from a previous dynamical model for ^222^Rn and soil moisture content in the Altamira cave by Sáez et al.^9^.

The Altamira cave exhibits a particular behaviour (the CO_2_ accumulates when the outside temperature is lower than the inside one), and a particular geomorphology. Moreover, it is located in a specific climatic area (where the soil moisture content plays an important role in the dynamics). Therefore, these models cannot be directly applied to other caves, although the technique can be transferred. Indeed, as the global modelling technique is a data-driven approach that requires no assumptions about the physical processes taking place in the system, the technique would be applied exactly in the same way, although the resulting models and their interpretations may be different and they might require to include other variables. For example, in locations with an active hydrological system connected to the karstic environment, significant aspects of the groundwater in the unsaturated zone should be considered such as the buffering effect of carbonate aquifers, or CO_2_ inputs from the groundwater aquifer to the unsaturated zone.The latter may be particularly relevant in geologically and geothermally active zones^49^.

### Reconstruction of CO_2_ concentration using in situ data as forcing

The original dynamics of the cave micro-atmosphere—not only the model—being itself highly sensitive to the initial conditions, any forecasts will diverge exponentially from the corresponding observed trajectories starting from any observed initial conditions. In practice, in order to get realistic simulations of the seasonal cycles of CO_2_ concentration, the information provided by the observed drivers should be used as forcing.

This can be done by reformulating the original autonomous system in a non autonomous one **M**_C_^*^ by replacing the variables *T* and *W* in model **M**_*CTW*_ by a forcing explicitly function of time, that is, by using the observed time series of surface temperature and soil water content to drive the model (see Supplementary Note 3 for details). In practice, this was done by rewriting model **M**_*CTW*_ (Eqs. 15–20) as

2 $$\dot{C} = a_{0} + P_{C} \left( {T_{{{\text{obs}}}} (t),\dot{T}_{{{\text{obs}}}} (t),W_{{{\text{obs}}}} (t)} \right) - a_{9} C_{{{\text{obs}}}} W_{{{\text{obs}}}}^{{2}} (t),$$

in which *T*, $\dot{T}$, and *W* have been replaced by the observational time series *T*_obs_(*t*), $\dot{T}_{{{\text{obs}}}} (t)$, and *W*_obs_(*t*) now used as external forcing, explicitly function of time *t*.

**Figure 4:**
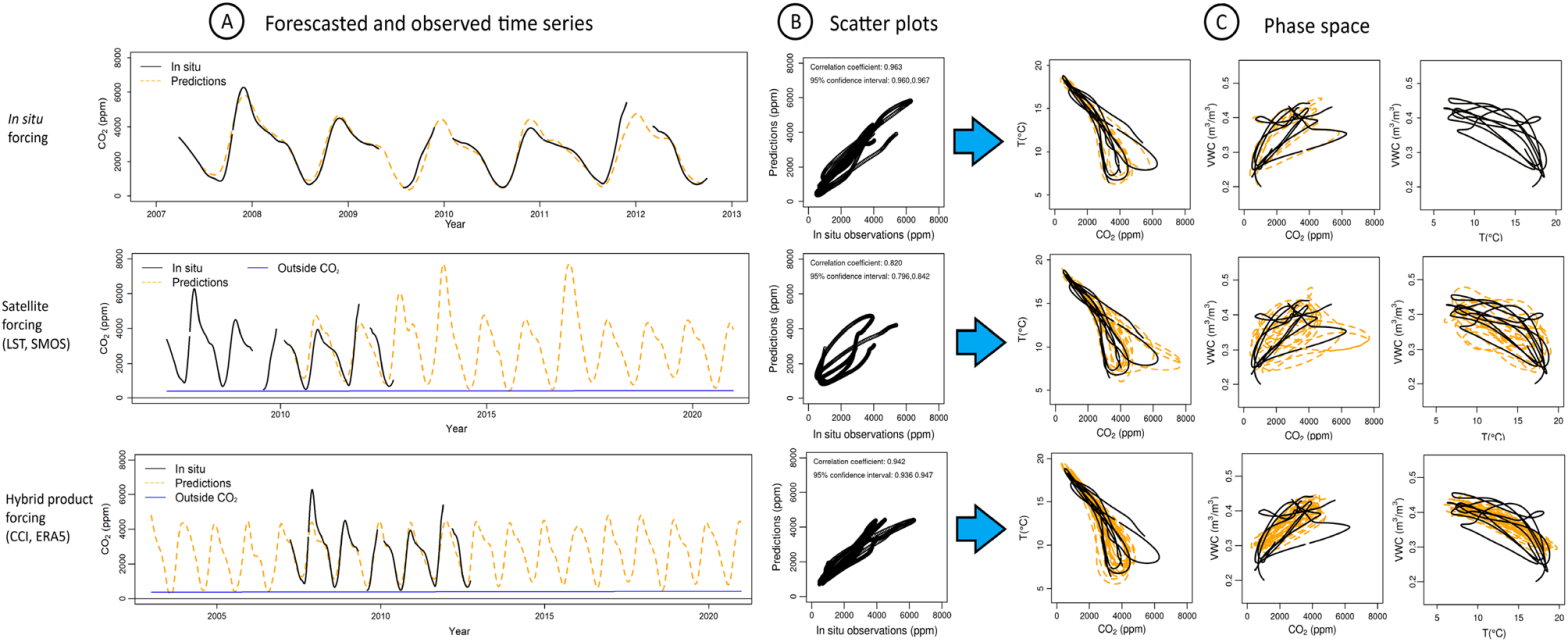
Non-autonomous modelling of CO_2_ concentration in the cave. Predictions are based on the non autonomous model **M**_C_^*^, considering either in situ forcing (top row), satellite forcing (middle row) or hybrid product forcing (bottom row). Original (plain line) and simulated (dashed lines with its associated error in light grey envelope) time series are provided in (**A**), modelled versus observed scatter plots in (**B**), and original (plain line) and model (dashed line) phase space reconstructions in (**C**).

The temperature *T* and soil moisture *W* forcings, taken from observed conditions in situ*,* enable to maintain the seasonal synchronization between the micro-atmosphere dynamics and the outside atmosphere, which is a common problem when autonomous equations are used. The synchronization being now maintained by the genuine forcing, all the conditions are gathered for validating the simulations: the modelled concentration of CO_2_ can be directly compared to the time evolution observed in the cave. The model concentration *C* shows a high degree of correlation (0.964, confidence level 95%) with the CO_2_ time series observed in situ (see Fig. 4A,B top line). The dynamics of the CO_2_ is thus reproduced correctly, and quite realistic simulations can be generated based on valid drivers. Moreover, the dynamics of CO_2_ is properly reproduced as illustrated by the various projections of the phase space (Fig. 4C top line), which mimics correctly the shape and size of the original orbits and also occupies a wide region of the original phase space.

The present model provides strong evidence of the combined roles of temperature and soil water content as main drivers of the dynamics of the cave micro-atmosphere.

### Recent decades of CO_2_ concentration reconstructed using satellite data

Although the in situ monitoring is 5 year long, it cannot be used to reconstruct the evolution of CO_2_ in the cave in the long term. Other in situ measurements are rare, not available in the long term, and even quite punctual when available. Thanks to model **M**_C_^*^ (Eq. 2), as just shown, valuable simulations of the concentration of CO_2_ can now be performed based on external observations. Satellite data might thus also serve for this objective. Different remote sensed products were then tested to run the model on longer periods of time. The measurements performed from space being, for the most, based on electromagnetic signals, they can only provide indirect information about the physical variables measured at the surface of the Earth. Moreover, satellite measurements are performed with specific space and time sampling. For these reasons, a dedicated pre-processing (recalibration, re-sampling and filtering) was required to make them compatible with the model input, the characteristics of which rely on the in situ measurements from which the model was derived (see Supplementary. Note 2 and Supplementary. Table 1, 2, 3). A similar type of pre-processing was applied to all the products used as a forcing in order to keep the consistency with the original variables.

The first mission dedicated to the measurement of soil moisture from space is SMOS, a European (ESA/CNES/CDTI) mission, initiated at the *Centre d’Etudes Spatiales de la Biosphère*^50^ and launched in 2009. It has produced measurements of soil moisture since the early 2010s and up to now. Data are considered here on the period 2010–2020. Based on ancient satellite measurements originally dedicated to other objectives, algorithms have also been developed to estimate soil moisture a posteriori. Two other products, both built from an ensemble of satellite missions, were considered in the present study: the Earth System Model (ESM) Soil Moisture product^51^ for which time series were considered on the period 1970–2016, and the ESA-CCI Soil Moisture data record^52^ from which the recent period 2003–2020 of the data was used here.

Temperature, also required to run model **M**_C_^*^, can be derived from remote sensing as well. Land Surface Temperature can be deduced from the thermal infra-red brightness temperature, commonly measured from satellite in the optic bands. For the present purpose, the MODIS satellite product available since the early 2000s^53^ was used. To go back further in the past, the ERA5 temperature at 2 m reanalyses product of the *European Centre for Medium-range Weather Forecasts* (ECMWF) was also used when necessary (down to 1950)^54^. The overlapping time of these various products with the in situ measurements are synthesized in Supplementary Table 2, 3.

The satellite forcings allow a notable reconstruction of the phase space. The phase space projections for CO_2_ concentration and temperature are shown in Fig. 4 (middle and bottom lines). The petal shape of the original orbits is retrieved correctly, as well as the general range of the variables. The combinations of forcings including systematically satellite data (SMOS + MODIS, ESM + MODIS, CCI + MODIS) or partially based on it (SMOS + ERA5, CCI + ERA5), enabled to produce very realistic simulations of CO_2_ concentration in the cave during the last two decades.

In conclusion, the multivariate model was initially based on in situ measurements, however these are available only on limited periods of time and often include gaps because caves are humid environments quite aggressive for the measuring devices. The use of satellite data for the temperature and the water content leads to some advances. They made it possible to approximate the dynamics of the CO_2_ over time periods for which no in situ measurements were available. In the next sections, we will use the model to investigate the impact that climate change may have in the future, and to explore the overwhelming effects of the massive touristic visits in the past.

### Future evolution of the CO_2_ concentration in the cave under climate change scenarios

Another concern is the future concentration of CO_2_ in the years and decades to come. What will be the effect of climate changes on the CO_2_ concentration in the cave and on its capacity to act as a source or sink of CO_2_?

To investigate these questions, simulations were performed based on scenarios of the global circulation model ECHAM4 produced by the Intergovernmental Panel on Climate Change (IPCC), run with observed conditions taken from the ECMWF on the period 1860–1990^55^. Six scenarios were produced by the IPCC based on four different story lines, one of them including several sub-scenarios. Four of the six scenarios were considered in the present analysis, that is, one for each main storyline. Scenarios A1 and B1 both assume an increase of the world population that will peak by mid-century, followed by a decline. The scenario A1 was built on the hypothesis of quick economic growth with rapid introduction of more efficient new technologies, high capacity building and increasing interactions leading to a reduction in regional inequities. Among the sub-scenarios of A1 produced by the IPCC, the present study focuses on the scenario A1B assuming balance across all sources of energy with similar improvement rates in supply and end use on the possible sources. Scenario B1 assumes a reduction of material intensity in the economic structure with the introduction of clean and efficient technologies, global solutions to sustainability and improved equity. The scenario A2 was built with the assumption of a very heterogeneous world preserving the local identities, with continuous increase of the populations and slow economic growth and technology change. The last scenario, B2, was built on the assumption of a world emphasizing local solutions to economic, social and environmental sustainability, also with a continuous population growth but with a lower increasing rate than that of A2. Although the products corresponding to the various scenarios were all issued from the same website, these were characterized by different spatial resolution (See Supplementary Note 2).

At the study site, the temperature increase by the end of the century is of + 3.5 °C for A2, + 3 °C for A1B, + 2.5 °C for B2 and + 2 °C for B1. The scenarios A2 and B2 also exhibit a progressive increase of the variables ranges, that is, higher maxima and lower minima. A slight soil moisture decrease is expected for all scenarios although it is more pronounced for A2 (− 0.05m^3^/m^3^) and B2 (− 0.03m^3^/m^3^) than it is for A1B and B1 (− 0.01m^3^/m^3^). The soil moisture for scenarios B2 and A2 was found quite coherent with the in situ data (available 2007–2012). It was not the case for the scenarios A1B and B1 (see Supplementary Table 2) whose soil moisture exhibits a non stationary dynamics (see Supplementary Table 1). Therefore the latter scenarios might produce less reliable CO_2_ simulations for Altamira.

The model **M**_*C*TW_, and thus its autonomous version **M**_C_^*^, was extracted from a short observational time series (2007–2012, including the modelling and the validation windows). Consequently, it reflects implicitly a stationary dynamics of the CO_2_ concentration in the outside atmosphere (assuming that the slow evolution of the climate is undetectable on a 5 year window). However, for long term predictions, the change predicted by the IPCC in the outside CO_2_ concentration should be considered. In particular, the level of CO_2_ in the cave should never become lower than in the outside atmosphere. For this purpose, the model was reformulated into **M**_C_^**^ as

3 $$\dot{C} = a_{0} + {\Lambda }_{Atm} \left( {C^{ext} \left( t \right) - C_{2007}^{ext} } \right) + P_{C} \left( {T_{obs} \left( t \right),\dot{T}_{obs} \left( t \right),W_{obs} \left( t \right)} \right) - a_{9} C_{obs} W_{obs}^{2} \left( t \right),$$

with C^ext^(*t*) the CO_2_ concentration observed or expected in the outside atmosphere, C^ext^_2007_ the value of C^ext^(*t*) in the year 2007, and $\Lambda_{Atm}$ a constant parameter which was calibrated to the value + 0.20 to satisfy this condition (see Supplementary Note 5 for details).

**Figure 5:**
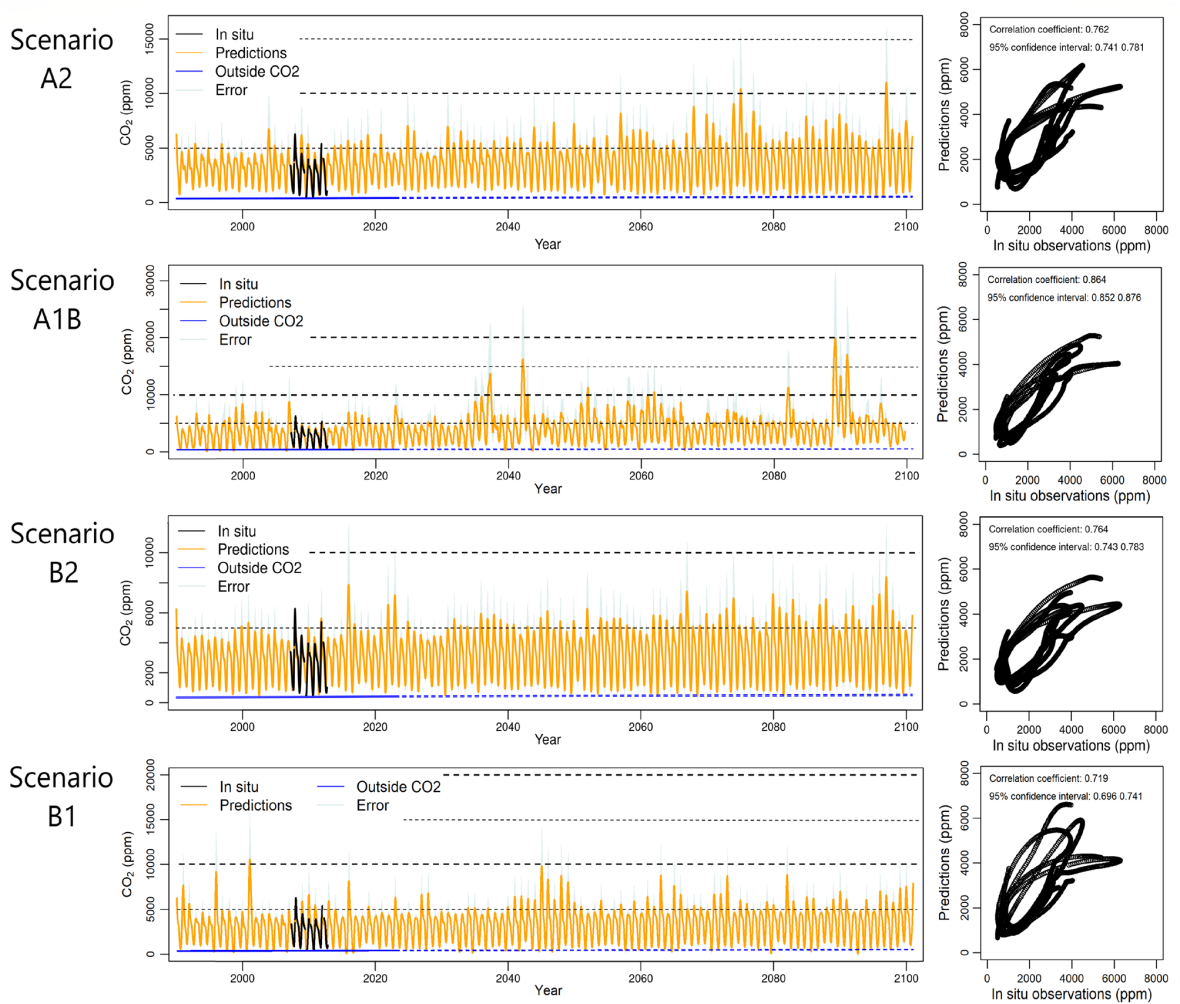
Future scenarios of CO_2_ in the Altamira cave (from 1990 to 2100). Reconstruction of the concentration of CO_2_ in the cave micro-atmosphere considering four IPCC scenarios used as forcing. Scenario A2 (first row) corresponds to a local increase of temperature of + 3.5 °C by the end of the century; Scenario A1B (second row) to a local increase of + 3 °C; Scenario B2 (third row) to a local increase of + 2.5 °C and Scenario B1 (fourth row) to a local increase of + 2 °C only. In all the cases, concentrations measured in situ are reported in black lines in (**A**) and used for the scatter plots in (**B**). Observations from 1990 to 2022 (blue plain line) and linear extrapolation from 2023 to 2100 (blue dashed line) of the CO_2_ outside the cave are also provided. Levels corresponding to 5,000 ppm, 10,000 ppm, 15,000 ppm and 20,000 ppm (when visible) are reported on the graphs in order to make the scenario comparison easier.

The Earth atmosphere being chaotic, each scenario must be considered as one particular realization of the given scenarios. Nevertheless, these can be used to study the general trends, and the possible evolutions of the distribution of the CO_2_ concentration inside the cave. Figure 5 shows that, while the minima remain essentially unchanged, the mean CO_2_ concentration in the cave raises approximately 1000 ppm for scenarios A1B and A2 by the end of the century, from 3000 to 4000 ppm; for the scenarios B1 and B2 the increase is somewhat lower (800 ppm), as expected from their lower increase of mean temperature. In addition to the increase of mean CO_2_ concentration, the scenario A1B shows a few occurrences of very extreme annual maxima. These outliers, which start mid-century, probably result from the poor representativeness of the IPCC soil moisture content time series, which therefore cannot reflect accurately the climate of Altamira. The average annual maximum of outside temperature and CO_2_ concentration rise slightly for the B2 scenario and quite notably for the A2 scenario, as well as the frequency of occurrence of larger than usual CO_2_ maxima (annual maxima up to 7000–9000 ppm are often forecasted from 2070 onwards). An increased range of the outside temperature will lead to sharper temperature gradients between the outside and the inside. These temperature gradients have important consequences when daily and short-term temperature oscillations are considered. In the present day, Altamira is located in an area where the annual temperature range is moderate, thus daily and short-term temperature oscillations can quite easily cause short periods of ventilation in winter and stagnation in summer. Therefore, the cave is neither completely closed in winter, nor completely open in summer. An increased outside temperature range might lead to a complete or more complete stagnation in winter and ventilation in summer, and thus, to increased CO_2_ accumulation in winter and ventilation in summer, as the simulations predict and as observed in caves located in other climatic areas^56,57^. Climate changes are also expected to affect the production of CO_2_ in the soil. In principle, high soil moisture and high temperatures foster the production of CO_2_, soil moisture being the most decisive factor^58^. In this case, an increase of temperature and a slight decrease of soil moisture are expected in the future. This is consistent with an increase of the CO_2_ production in the soil, especially in winter, and thus with the larger concentration of CO_2_ predicted in the cave during the charged state, when ventilation is inhibited. Finally, the higher concentration of CO_2_ expected by the IPCC in the outside atmosphere, might affect also the level of CO_2_ in the cave, especially in summer. The biological CO_2_ coming from the soil will be ventilated by air richer in atmospheric CO_2_, therefore the minimum possible CO_2_ value in the cave will be slightly higher. These estimates are very likely local since they are based on an ad hoc model built for the Altamira cave. At this stage, they can neither be generalized to soil around the cave, nor to other caves in general because the dynamics of caves can be very diverse depending on their detailed morphology and climatic location. To investigate the genericity of this result on the atmosphere, the present approach should be applied to other regions and other karstic and underground systems. However, in the inside of the Altamira cave, the enhanced accumulation of CO_2_ in its atmosphere and in the infiltrated water will increase the risk of micro-corrosion^15^. This is clearly a threat to the paintings because micro-corrosion partially removes the pigments and the rock surface where they lie. Indeed, the IPCC scenario that leads to larger impact for the cave CO_2_ concentration is scenario A2, which is also the most probable scenario considering the present evolution of technology, economy and population.

### Past evolution of the CO_2_ concentration under touristic regime

Land surface temperature and soil moisture measurements from satellite were not available before 2000. Despite this, it is possible to attempt a simulation of past scenarios based on climate data coming from reanalyses. The CO_2_ concentration in the cave was thus reconstructed back to 1970 by using the ESM and ERA5 products. Another reconstruction, exclusively based on the ERA5 product could be generated back to 1950. In addition to its natural dynamics, the Altamira cave has undergone different affluence regimes^59^, therefore different situations must be distinguished. Following a long period of increasing tourism activities from 1952 to the early 1970s, with a peak of 174,613 visits recorded in 1972, the Altamira cave closed its doors from 1978 to 1982 after noticing a rapid degradation of the Palaeolithic paintings. A museum with reproductions was opened to satisfy the touristic demand. The access to the site was restored but limited to a smaller number of visitors during the 1982–2002 period (11,320 visitors per year). In 2002, Altamira was closed for the second time to the public because of the presence of phototrophic micro-organisms on the paintings^8^, resulting from the presence of visitors and the exhalation of CO_2_, amongst other factors. However, touristic exploitation was resumed in 2014 although restricted to a very low number of visitors (5 per week) to ensure longer preservation. Reconstructions based on two scenario types were then performed to analyse this situation. In the first type, the influence of the visitors was excluded. For this purpose, model **M**_C_^**^ presented in Eq. (3) was taken as it was obtained (since built on a period free of visitors). In the second type, the model was reformulated into **M**_C_^***^ as

4 $$\dot{C} = a_{0} + {\Lambda }_{Atm} \left( {C^{ext} \left( t \right) - C_{2007}^{ext} } \right) + {\Lambda }_{Vis} V\left( t \right) + P_{C} \left( {T_{obs} \left( t \right),\dot{T}_{obs} \left( t \right),W_{obs} \left( t \right)} \right) - a_{9} C_{obs} W_{obs}^{2} \left( t \right),$$

to take the influence of the visitors into account, with V(t) the number of visitors per day at time t and $\Lambda_{Vis}$ a constant parameter (see Supplementary Note 5 for details). The in situ time series of CO_2_ concentration from 1997–1998^15^ was used to calibrate $\Lambda_{Vis}$, the additional contribution to CO_2_ concentration per visitor and per day.

**Figure 6:**
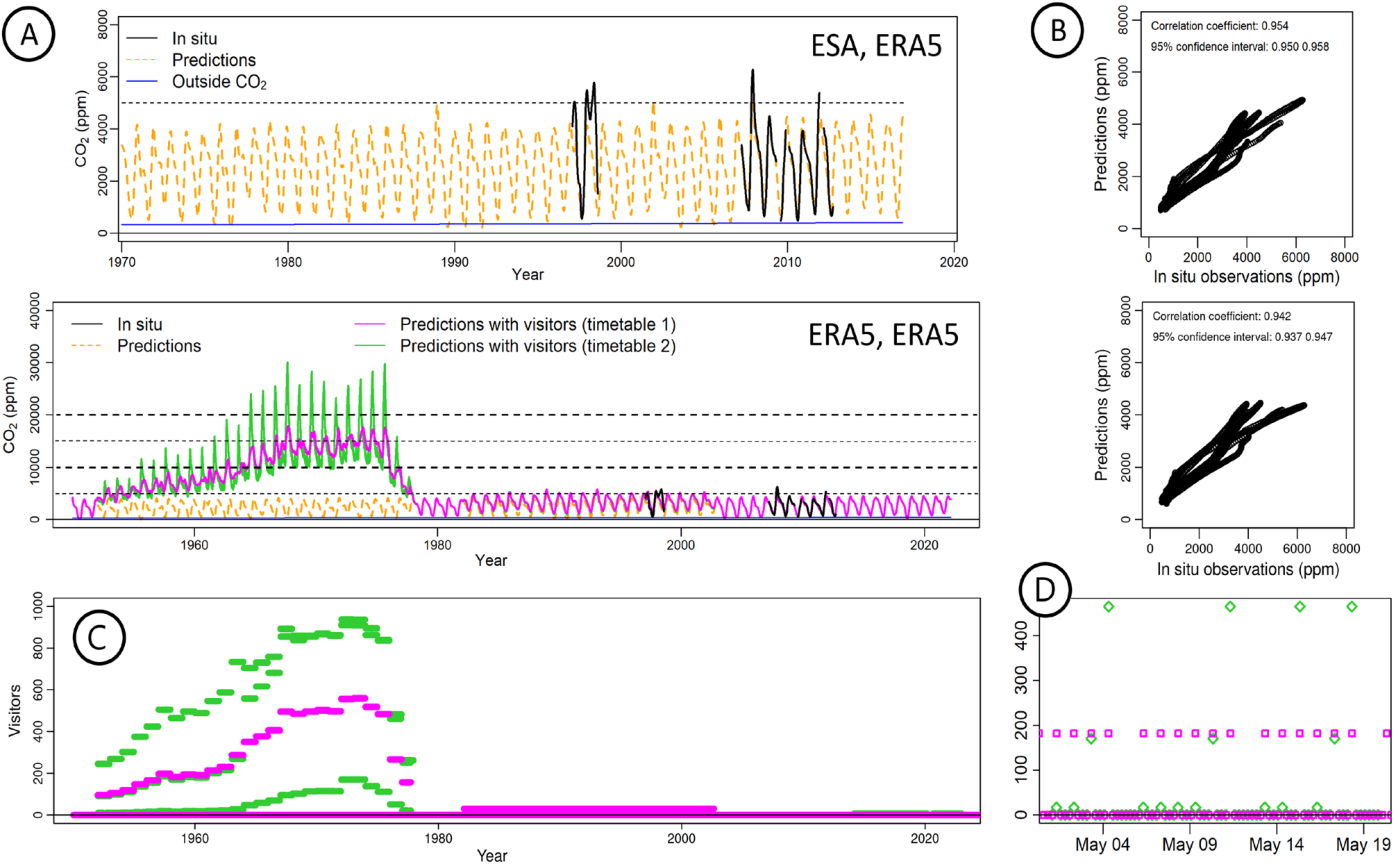
Past scenarios of CO_2_ in the Altamira cave (from 1950 to 2022). Reconstruction of the concentration of CO_2_ in the cave micro-atmosphere considering different scenarios. For the period 1970–2016 (**A**), the concentration is reconstructed (orange line) without visitors, using the ESA hybrid product for soil moisture and the ERA5 model for temperature. For the period 1950–2022 (**B**), the CO_2_ concentration is reconstructed using both the temperature and soil moisture data from ERA5: without visitors (orange dashed line), with yearly equally distributed affluence of visitors (magenta line) and with affluence of visitors depending on the festivity of the day (green line). The observed in situ data are reported in black, the CO_2_ outside the cave, in blue. The time series of the number of visit every 12 h is presented in (**C**) under the two hypotheses: equal-distribution of the yearly visits (pink line), realistic distribution of the yearly visits (green line). The original record of yearly visits is provided in Supplementary Table 11. In (**D**) a zoom of three weeks of (**C**) is presented as an example of the hypothesized daily visitors in 1956. The points in the zero level represent the nighttime (recovery time with no visitors) and the points over zero, the daytime. The magenta points indicate 200 visitors from Tuesday to Sunday and no visits on Monday. The green points indicate few visitors from Tuesday to Friday, approximately 200 visitors on Saturdays and more than 400 visitors on Sundays. Levels corresponding to 5,000 ppm, 10,000 ppm, 15,000 ppm and 20,000 ppm are reported on the graphs to facilitate the comparison with Fig. 5.

Without visitors pressure, the simulations of CO_2_ concentration reconstructed from 1950 to 2000 are very consistent in range with what was observed during the last two decades (Fig. 6). Two simulations with visitors were performed. For the first simulation (visits homogeneously distributed through the year), the CO_2_ predictions reach maximum values of almost 20,000 ppm during the 1970s. Taking the variations of the visits at daily, weekly and yearly scales into account for the second simulation reveals maxima values even higher (up to 30,000 ppm). In both cases the mean CO_2_ concentration reaches approximately 15,000 ppm in the 1970s. It is to be noted that peaks of high to very high concentrations of CO_2_ have been already observed in other caves: at Lascaux (15,000 ppm on average)^7^, at Comblain-au-Pont cave (up to 27,500 ppm)^60^ or at the Natural Bridge Cavers (up to 38,200 ppm)^61^. In summer the affluence of visitors to Altamira must have been particularly high during the whole day, strongly increasing the CO_2_ concentration; in addition, ventilation during the summer nights is limited because the soil membrane is blocked due to the adsorption and condensation of water on the surface^20^. Indeed, in the past, the entrance of visitors in Altamira produced local phenomena of air stagnation. Another probable factor contributing secondarily to the accumulation of CO_2_ in the cave was the low temperatures recorded during that period. The years from 1950 to 1985 displayed significant negative temperature anomalies, particularly pronounced during the summer months^62^. This decline in summer temperatures resulted in a notable reduction in ventilation rates, especially noticeable in the peaks of July and August, impeding air renewal and facilitating the gradual CO_2_ build-up in the underground environment.

Nevertheless, very punctually, the model seems to exaggerate the CO_2_ minima, although it is not possible to know to which extent. After drastic limitation of the entrances, the number of visitors reached zero in 1979. Its effect is clearly characterized by a drastic decrease of the CO_2_ concentration. The reopening with a limited number of visitors per year during the period 1982 to 2002 exhibits a clear shift of around + 730 ppm on average with annual maxima in the range 4400–5500 ppm, that is, significantly higher than the simulations without visitors (3800–4600 ppm). In spring 1998, a deviation of the predictions from the in situ measurements is observed (Fig. 6A). This is due to the affluence of workers in order to get detailed topographic data and photographic records for the construction of a replica of the cave (the *Neocueva*), which caused a cumulative effect on the CO_2_ concentration^63,64^. The observed deviation corresponds to these impacts, which were not included in the models, as the access regime to the cave during these works is unknown. Regarding the minimum values, they might seem overestimated, but our results are quite coherent with the difference in mean observed in the measured time series: 459 ppm larger in 1997–1998 (~ 11,320 Visitors/year) than it was in 2007–2012 (~ 0 Visitor/year). This effect occurs as a consequence of the entry of consecutive groups into the Polychrome Room, which generates cumulative disturbances in the internal microenvironment. Specifically, in the study carried out in 1997–98 it was found that from mid-July to the end of August the cumulative effects occurred continuously and the full recovery of the temperature and CO_2_ concentration was never achieved before the entry of the next cycle of daily visits (Supplementary Fig. 5 from Suppl. Online Mat. of^12^). An estimation of the micro-corrosion suffered by the cave in 1997 showed values 78 times greater than the theoretical value derived only from natural phenomena of water condensation on the walls^15^. In addition to the strong increase in the CO_2_ concentration, the entry of visitors into the cave entails a significant thermal rise and the emission of large amounts of water vapour that must have caused condensation and corrosion phenomena of great magnitude. Therefore, even if the number of visitors in this period (1982–2002) was quite restricted when compared to the past, the cave environment was still quite perturbed. Yet, the CO_2_ levels in that period (Fig. 6A) are lower than the levels predicted for the future, both in the A2 and B2 scenarios (Fig. 5), therefore climate changes to come in the next decades pose a clear threat to the Palaeolithic paintings.

## Conclusion

Extracting dynamic equations from time series is a growing field, but rarely applied to environmental problems. In this work, the global modelling technique was used to obtain sets of equations for the dynamics of air CO_2_ in the Altamira cave. The algebraic formulation of the obtained models confirmed the two main drivers of the micro-atmosphere dynamic: the surface temperature and the soil water content. Based on them, it was then proven possible to apply the obtained equations to quite new applications.

Thanks to these dynamical models, it was first shown possible to use satellite data to monitor the dynamics of CO_2_ in the cave, which was quite unexpected. In particular, estimates of soil moisture derived from the SMOS satellite and of land surface temperature from the MODIS mission were used to reconstruct the cycles of CO_2_ in the cave from 2010 to the present. In the past, in situ measurements were performed in the cave but during limited periods of time and often with gaps in the records since caves are humid environments that tend to damage the measuring devices. As a consequence, such measurements cannot be performed continuously on the long term. The use of satellite data may enable a useful advance for this monitoring purpose. Moreover, it opens the possibility of applying the approach to other caves and also to other molecules (Rn, water vapour, any tracers). It may also be used to study other topics related to the underground since the methodology is well suited to tackle with problems stemming from the difficulty of measuring the variables involved in the dynamics.

Another unexpected application that arose from the reconstructed equations was the possibility to generate scenarios of the cave CO_2_ concentration in the past, which was not possible before. Since the multivariate model was obtained for a period without visitors, only simulations free of visits may have been performed with the model as it was obtained. But as the model is of algebraic formulation, it was possible to analyse it and to reformulate the equations to account for the visitors’ exhalation of CO_2_, considering it as a direct anthropogenic forcing. Clearly, the obtained equations offer more than a black box and can thus be used to generate scenarios. The yearly number of visitors was used as a forcing for this purpose and multiple simulations were generated based on three scenarios: one free of visitors and two based on the historical dataset, assuming different regimes of visit distributions. The latter scenario enabled us to estimate the concentration of CO_2_ in the cave from the early 1950s and up to present, indicating the extremely high levels that were likely reached during the massive period of visits (until 1972) that fostered the development of micro-organisms endangering the prehistoric paintings. It revealed also the more moderate – but still too high for a sustainable situation—levels reached in the 1980s after the cave was reopened to the public. The scenario free of visitors is also very promising since it suggests the possibility of reconstructing the atmosphere conditions of the cave much earlier in historic and prehistoric times, which could be another great challenge for future investigations.

This having been done for the past, the will to investigate the future came naturally. But with the future of the cave comes the question of the future climate, which remains largely uncertain. This question was investigated considering the products of the IPCC as forcing. Although the products we used in the study were all considered equally probable in the early 2000s, only the ones with a quicker temperature increase remain possible today. The simulations of CO_2_ concentration in the cave corresponding to these scenarios suggest new risks for the future of the cave.

Equation reconstruction and chaos-based modelling techniques appear as a promising approach for investigating complex, open problems for which no equations are available. Such an approach is particularly promising since it may be used, even under limited variability of in situ observation, to monitor the present using satellite information, to reconstruct the past, and to help anticipating the future.

## Methods

### GPoM Algorithm and modelling framework

The Global Polynomial Modelling (GPoM, version 1.3) package^65^, developed in R language, was the main tool used for modelling. The GPoM algorithm aims to obtain ordinary differential equations (ODEs) from observational time series. To run the GPoM algorithm, a general structure is required. In practice, we strictly focused on polynomial structures in the present analysis.

When starting from a single time series *X*_1_^obs^(*t*), the numerical description of the ODEs canonical formulation is defined by

5 $$\left\{ {\begin{array}{*{20}c} {\dot{X}_{1} = X_{2}  \quad \quad \quad \quad \quad \quad \quad \quad \quad } \\ \begin{gathered} \dot{X}_{2} = X_{3} \quad \quad \quad 
 \quad \quad \quad \quad \quad \quad \hfill \\ \quad \, \vdots \hfill \\ \end{gathered} \\ {\dot{X}_{{n_{X} }} = U_{X} \left( {X_{1} ,X_{2} ,..,X_{{n_{X} }} } \right)\,,} \\ \end{array} } \right.$$

with *X*_1_ the observed variable, and *X*_2_ to *X*_*nX*_ its successive derivatives, *n*_*X*_ the model dimension and **U**_X_ a function to be fully retrieved, both in its structure and parametrization^27^. This formulation requires fixing the following parameters: (i) the model dimension *n* (that is, the number of derivatives to be used, including the original time series without derivation) and (ii) the maximum polynomial degree *q*^max^. The algorithm will be run eight times: one for each combination of *n* = 3–4 with *q*^max^ = 2–5.

When starting from a set of *N* observational time series (*X*_1_^obs^, *Y*_1_^obs^ … *Z*_1_^obs^), the following mathematical formulation is used:

6 $$\left\{ {\begin{array}{*{20}l} {\dot{X}_{i} \; = \;X_{i + 1} \quad {\text{for}}\;i = 1\;..\;\left( {n_{X} - 1} \right) \quad \quad \quad \quad \quad \quad \quad \quad \quad \quad \quad \quad \quad \;} \\ \begin{gathered} \dot{X}_{{n_{X} }} = F_{X} \left( {X_{1} ,X_{2} ,\;..\;,X_{{n_{X} }} ,Y_{1} ,Y_{2} ,\;..\;,Y_{{n_{Y} }} ,\;...\;,Z_{1} ,Z_{2} ,\;..\;,Z_{{n_{Z} }} } \right)\; \hfill \\ \dot{Y}_{i} \;\; = \;\;\dot{Y}_{i + 1} \quad {\text{for}}\;i = 1\;..\;\left( {n_{Y} - 1} \right)\quad \quad \quad \quad \quad \quad \quad \quad \quad \quad \hfill \\ \dot{Y}_{{n_{Y} }} \;\; = F_{Y} \left( {X_{1} ,X_{2} ,\;..\;,X_{{n_{X} }} ,Y_{1} ,Y_{2} ,\;..\;,Y_{{n_{Y} }} ,\;...\;,Z_{1} ,Z_{2} ,\;..\;,Z_{{n_{Z} }} } \right)\; \hfill \\ \quad \;\;\,\, \vdots \hfill \\ \end{gathered} \\ \begin{gathered} \dot{Z}_{i} \; = \;Z_{i + 1} \quad {\text{for}}\;i = 1\;..\;\left( {n_{Z} - 1} \right)\quad \quad \quad \quad \quad \quad \quad \quad \quad \quad \hfill \\ \dot{Z}_{{n_{Z} }} \;\; = F_{Z} \left( {X_{1} ,X_{2} ,\;..\;,X_{{n_{X} }} ,Y_{1} ,Y_{2} ,\;..\;,Y_{{n_{Y} }} ,\;...\;,Z_{1} ,Z_{2} ,\;..\;,Z_{{n_{Z} }} } \right)\,, \hfill \\ \end{gathered} \\ \end{array} } \right.$$

with (*X*_1_, *Y*_1_ …*Z*_1_) the observed variables and (*X*_*j*_, *Y*_*k*_ … *Z*_*l*_) their successive derivatives, *n*_*X*_, *n*_*Y*_ … *n*_*Z*_, the number of variables effectively used for each observational time series, and *F*_*X*_, *F*_*Y*_ … *F*_*Z*_ the functions of which both structure and parameters have to be identified. This structure can be described numerically by fixing the number of derivations to be used for each time series, and, again, the maximum polynomial degree *q*^max^. More specific formulations may also be used (typically, to make the computation assessable by avoiding some unrealistic coupling between the variables). Each of these formulations requires a dedicated run of the algorithm.

For a given general structure, the ensemble of possible models can be absolutely tremendous in some cases (see Supplementary Note 4). The GPoM algorithm has been proven to be very powerful in reducing drastically the number of possible models to be explored, in order to retrieve the original formulation when it exists, or to get a good approximation of it when it does not^33,36,66^. The modelling protocol applied to each general structure can be summarized as follows. The GPoM algorithm works in two stages: the first stage aims to reduce drastically the number of candidate models; the second stage aims to test the robustness of the preselected candidates. The following inputs are required: Single or multiple time series with *t* the same discrete values of time with regular sampling; The number of variables (*n*_*x*_, *n*_*y*_, …) derived from each time series; The window length used to compute the derivatives (by default 9 points are used); And the maximum polynomial degree *q*^max^ allowed in the general numerical formulation. Optionally, a more specific general structure can be designed by hindering some couplings, limiting the total number of terms *N*_P_, or limiting the number of terms for a given equation, etc.

The structure selection algorithm aims to reduce drastically the number of candidate models without strong a priori hypotheses about the size of the model. At this stage, the equations are considered one by one, separately. For a given equation, all the *p* terms of the general equation structure are considered at the very beginning, subsequently some of them will be discarded as follows. (1) A Gram-Schmit procedure is used to calculate the equation parameter values. (2) A leave-one-out technique is applied to estimate the influence of each term and to find the term of minimal influence on the residual signal. The distance

7 $$d = \sqrt {\frac{1}{N}\sum\limits_{j = 1}^{N} {\left( {\dot{X}_{{n_{X} }}^{obs} (t_{j} ) - \hat{P}\left( {X_{1}^{obs} (t_{j} ),X_{2}^{obs} (t_{j} )\;...\;X_{{n_{X} }}^{obs} (t_{j} )} \right)} \right)^{2} } }$$

is used for this purpose, with *N* the time series length. (3) The term of minimal influence is removed and a substructure of (*p−*1) terms is obtained. Operations (1) to (3) are repeated until all the terms are removed. The general formulation, which enabled initially 2^*p*^ particular formulations, is now reduced to (*p* + 1) ones. By applying the same algorithm to each equation, the algorithm enables to reduce drastically the number of original formulations from 2^(*px*+*py*+ …+*pz* )^ to (*p*_*x*_ + 1) × (*p*_*y*_ + 1) × … (*p*_*z*_ + 1), where *p*_*x*_, *p*_*y*_ … *p*_*z*_ are the number of terms to be investigated for each equation in the original general formulation. The algorithm is described with further details in^33^ where its robustness is also tested and discussed.

The selection algorithm yields a number of preselected models. Then, the preselected models are tested by numerical integration on duration sufficiently long to get out from the transient. More practically, this duration should be as long as possible, that is, until the attractor is reached, in order to have a proper description of the underlying dynamics^46^. Divergent models are rejected and the other ones are classified depending on their level of dynamical complexity, considering the attractor reached at the convergence as: fixed point (simplest dynamic), period-1 cycle, period-2 cycle, etc., and quasi-periodic attractor. Models presenting more complex dynamics (phase-coherent chaos, phase non-coherent chaos and hyper-chaos) are kept as unclassified since automatic detection is more difficult in these cases. Although presenting variations around an annual cycle, the dynamics of caves micro-atmosphere are obviously neither periodic nor quasi-periodic, and even less probably convergent to a fixed point. For this reason, these types of models are rejected and only the ones presenting a higher level of complexity are kept, for a more in-depth analysis. After these successive selection criteria are applied, only a very small set of models generally remains (as it was the case here) and the models obtained from all the general structures can be considered one by one, and compared. Note that just fitting the particular trajectory of the observations in the time space is not an appropriate choice since it can lead, almost surely, to an ad hoc model due to over parametrization. Therefore, several characteristics are used to choose the best models among the remaining ones, in terms of range of the variables, and density distribution. This selection enables to get global models, that is, models able to reproduce the global behaviour of the observed system. Final model validation and selection is then based on the forecasting performances. For models presenting equivalent performances, the model of simpler formulation will be preferred, following the principle of parsimony. Indeed, applied at the end of the selection process, this principle (Occam’s razor) has proven to be very powerful to retrieve original equations of compact form^33^.

The structural parameters of the global models considered in the present study (*n*_C_, *n*_T_, *n*_W_, *q*_max_) together with the other pre- and post- processing parameters are synthesized in Supplementary Table 4. The general structures are provided in this section (Eqs. 8–14).

### Univariate models

No specific templates were used or tested for modelling the dynamics from univariate time series (to get the models **U**_C_, **U**_*T*_ or **U**_*W*_) because the number of possible models was manageable considering the general formulation (see Supplementary Note 4). These were obtained by using the very general structure (Eq. 5), tested under dimension three to four and under polynomial degree two to five (*n* = 3–4, *q*^max^ = 2–5). For CO_2_ concentration *C*, the in situ time series was used on the period 2007–2012 (see Supplementary Note 2). For temperature *T*, a daily time series of land surface temperature issued from MODIS (MOD11C3) satellite product was used (The footprint of these datasets is shown on Fig. 1D); this product is available on the period February 2000 to June 2021. For soil moisture *W*, a specific reprocessing of the data issued from the *Soil Moisture Ocean Salinity* (SMOS) satellite was used from April 2010 to December 2020 (footprint shown on Fig. 1D); pre-processing details are provided in Supplementary Note 2. The modelling was applied following the procedure described in present section, as illustrated in Supplementary Fig. 2 (see^33^ for a discussion). Details about the obtained models are provided in Supplementary Note 3.

### Multivariate models

For the multivariate models, the total number of terms had to be limited in order to get affordable computation and processing times. Three and four dimensional models were tested, with maximum polynomial degree up to 3. A feedback from the cave micro-atmosphere (variable *C*) onto the outside atmosphere (variable *T*), and on the water content in the soil (variable *W*) cannot be excluded locally, but it can be assumed to be of secondary order. In the same way, a retroaction of the water content of the soil onto the outside atmosphere can be disregarded. The removal of these retroactions reduced the total amount of possible models based on each general structure. Therefore, the following general structures were tested:

8 $$\left\{ {\begin{array}{*{20}l} {\dot{C} = P_{C} \left( {C,W,T} \right)} \quad \\ {\dot{T} = P_{T} \left( T \right)\;\;\;\;\;\;\;\;\;} \\ {\dot{W} = P_{W} \left( {W,T} \right)\;\;\;} \\ \end{array} } \right.$$

with *q*^max^ = 2 (*N*_mod_ = 2^19^) to 3 (*N*_mod_ = 2^30^),

9 $$\left\{ {\begin{array}{*{20}c} {\dot{C} = C_{2} \quad \quad \quad \quad \quad \quad \;\;} \\ {\dot{C}_{2} = P_{C} \left( {C,C_{2} ,T,W} \right)} \\ {\dot{T} = P_{T} \left( T \right)\quad \quad \;\;\;\;\;\;\;\;\;} \\ {\dot{W} = P_{W} \left( {T,W} \right)\quad \quad \;\;} \\ \end{array} } \right.$$

with *q*^max^ = 2 (*N*_mod_ = 2^24^) to 3 (*N*_mod_ = 2^49^),

10 $$\left\{ {\begin{array}{*{20}c} {\dot{C} = P_{C} \left( {C,T,W} \right)\;} \\ {\dot{T} = T_{2} \quad \quad \quad \;\;\;\;\;\;} \\ {\dot{T}_{2} = P_{T} \left( {T,T_{2} } \right)\;\;\;\;} \\ {\dot{W} = P_{W} \left( {T,W} \right) \;\;\;} \\ \end{array} } \right.$$

with *q*^max^ = 2 (*N*_mod_ = 2^22^) to 3 (*N*_mod_ = 2^40^),

11 $$\left\{ {\begin{array}{*{20}l} {\dot{C} = P_{C} \left( {C,T,W} \right)\quad \;} \\ {\dot{T} = P_{T} \left( T \right)\quad \quad \;\;\;\;\;\;\;} \\ {\dot{W} = W_{2} \quad \quad \quad \quad \;\;\;\;} \\ {\dot{W}_{2} = P_{W} \left( {T,W,W_{2} } \right)\;} \\ \end{array} } \right.$$

with *q*^max^ = 2 (*N*_mod_ = 2^23^) to 3 (*N*_mod_ = 2^44^), which remains more manageable, and in which it is also assumed that the second derivatives can have an action, only on the variable from which it is derived. Note that univariate models obtained from *C*, *T* and *W* were all three-dimensional, which is a required condition to get a minimum level of complexity (Poincaré-Bendixson theorem). Extending these latter four templates (Eqs. 12–15) by considering that at least one variable should be explicitly described by three dimensions, we get:

12 $$\left\{ {\begin{array}{*{20}l} {\dot{C} = C_{2} \quad \quad \quad \quad \quad \;\;\;\;\;\;\;\;\;\;\;} \\ {\dot{C}_{2} = C_{3} \quad \quad \quad \quad \quad \;\;\;\;\;\;\;\;\;\;} \\ {\dot{C}_{3} = P_{C} \left( {C,C_{2} ,C_{3} ,T,W} \right)} \\ {\dot{T} = P_{T} \left( T \right)\quad \quad \quad \;\;\;\;\;\;\;\;\;\;\;} \\ {\dot{W} = P_{W} \left( {T,W} \right)\quad \quad \quad \;\;\;\;} \\ \end{array} } \right.$$

with *q*^max^ = 2 (*N*_mod_ = 2^30^) to 3 (*N*_mod_ = 2^70^),

13 $$\left\{ {\begin{array}{*{20}l} {\dot{C} = P_{C} \left( {C,T,W} \right)\quad \;\;} \\ {\dot{T} = T_{2} \quad \quad \quad \quad \quad \quad \;} \\ {\dot{T}_{2} = T_{3} \quad \quad \quad \quad \quad \;\;\;\;} \\ {\dot{T}_{3} = P_{T} \left( {T,T_{2} ,T_{3} } \right)\;\;\;\;\;} \\ {\dot{W} = P_{W} \left( {T,W} \right)\quad \quad \;} \\ \end{array} } \right.$$

with *q*^max^ = 2 (*N*_mod_ = 2^26^) to 3 (*N*_mod_ = 2^50^),

14 $$\left\{ {\begin{array}{*{20}l} {\dot{C} = P_{C} \left( {C,T,W} \right)\quad \quad \quad \;\;\;} \\ {\dot{T} = P_{T} \left( T \right)\quad \quad \quad \quad \quad \;\;\;\;\;} \\ {\dot{W} = W_{2} \quad \quad \quad \quad \quad \quad \quad \;\;\;} \\ {\dot{W}_{2} = W_{3} \quad \quad \quad \quad \quad \quad \quad \;\;} \\ {\dot{W}_{3} = P_{W} \left( {T,W,W_{2} ,W_{3} } \right)\;} \\ \end{array} } \right.$$

with *q*^max^ = 2 (*N*_mod_ = 2^28^) to 3 (*N*_mod_ = 2^59^). These two latter templates could also be tested considering that the univariate parts were known (models of higher polynomial degree **U**_*C*_ and **U**_*T*_), and thus limiting the research to two polynomials only. The analysis was performed on the period 20th June 2007 to 26th September 2012, for which continuous in situ measurements of CO_2_, temperature and soil moisture inside and above the cave, respectively were available. Almost all the multivariate models selected from these templates (Eqs. 6–14) were rejected either directly, or at the validation stage. Only two multivariate models could pass the selection procedure before validation and only one could reach the end of the validation process: model **M**_*CTW0*_. This model follows the template given by Eqs. (10). It was able to reproduce the dynamics of temperature, volume water content and CO_2_ concentration but only in the short-term because the forcing was not stable enough for long-term integration. Therefore, the temperature and water content forcing was improved using the satellite data to get a model M_*TW*_ (see Supplementary Table 4), following an analogous process of search and validation. M_*TW*_ is a bivariate five-dimensional model for temperature and water content, following the structure showed in Eqs. (16–20), where Eqs. (16–18) correspond, in turn, to the univariate model U_*T*_. In summary, the model **M**_*CTW*_, which is able to reproduce the dynamics of the variables in the long term, was obtained by joining the first equation of model **M**_*CTW0*_ and model **M**_*TW*_, therefore it has the following structure

15 $$\dot{C} = a_{0} + P_{C} \left( {C,T,T_{2} ,W} \right)$$

16 $$\dot{T} = T_{2}$$

17 $$\dot{T}_{2} = T_{3}$$

18 $$\dot{T}_{3} = U_{T} \left( {T,T_{2} ,T_{3} } \right)$$

19 $$\dot{W} = W_{2}$$

20 $$\dot{W}_{2} = P_{W} \left( {T,W,W_{2} } \right)\,,$$

with *P*_*C*_, *U*_*T*_ and *P*_*W*_ three polynomials whose detailed definition is provided in Supplementary Note 3.

### Model error

Various sources can contribute to the error of the simulations: (i) the measurement errors for each in situ and satellite variable; (ii) the scaling error for the satellite data; (iii) the modelling error attached to the calibration of each coefficient of the polynomials; and (iv) the modelling error due to the fact that any model is inevitably a simplified version of reality. These errors are not independent. The following method was used to estimate their combined contribution. For the simulations with in situ forcings, the error was estimated as the rmse between the observations and the predictions on the calibration period because this error integrates most of the error sources. For the other simulations, the forcings were perturbed by adding ± 0.5 ºC to the temperature forcing and ± 0.025 m^3^/m^3^ to the VWC forcing, obtaining four CO_2_ simulations. From the four simulations, we extracted a time series with the minimum CO_2_ prediction at each time, in order to get a lower bar for the error. Similarly, the upper bar consists of the time series of maximum predictions. These estimates correspond to an intermediate level of error. Another source of error (v) should be mentioned: the error stemming from chaotic behaviour, which implies that small inaccuracies in the initial conditions lead to exponential divergence between the observed and predicted trajectories. This error source may result from the outside atmosphere used as external forcing, which is highly sensitive to the initial conditions as already mentioned upper in the text. This error was not estimated because a single product was made available for each scenario by the IPCC. This is not really a problem as far as scenarios are concerned. Indeed, scenarios are not forecasting, their role is to provide simulations of possible futures, not of the future itself. Each scenario is thus a particular simulation that will never occur as so, but that may reflect salient features of the future based on the chosen hypothesis. They can be used to investigate possible futures in terms of trends and statistics. The cave micro-atmosphere dynamics may also have shown a certain sensitivity to the initial conditions. In the present case, it was found that the cave micro-atmosphere was not chaotic in itself, and that it was not required to account for this error separately from the other contributions (i) to (iv).

### Models validation

To validate a global model is a difficult problem as far as chaotic dynamics are concerned. Indeed, even if the model is perfect, any error should give rise to a divergence between the model and the observation since the original dynamics is highly sensitive to the initial conditions. Nonlinear invariants are an option—in principle—but these are difficult to extract from observational—and thus noisy—short-length time series. For this reason, it is preferred to consider the forecasting capacities to validate the models. The model error growth

21 $$\left| {e_{\tau } } \right| = \left| {\frac{{\hat{X}_{t} \left( \tau \right) - X_{t + \tau }^{{{\text{obs}}}} }}{{X_{t + \tau }^{{{\text{obs}}}} }}} \right|,$$

was thus used for this purpose, with $\hat{X}_{t} \left( \tau \right)$ the forecasted prediction performed at time *t* for a horizon time *τ*, and $X_{t + \tau }^{{{\text{obs}}}}$ the observed value at the corresponding time *t* + *τ*. To characterize the error growth, ensemble of simulations were generated. These were estimated first on the modelling window, then on the validation window in order to estimate the degradation of the model on an independent data set. These ensembles were used to estimate the error growth with a confidence level of 90%, from which a horizon of predictability was also deduced considering a threshold of 5% relative error. The error growth obtained for models **U**_*C*_, **U**_*T*_, **U**_*W*_ and **M**_*CTW*_ is shown in Supplementary Fig. 3 and synthesized in Supplementary Tables 8 and 10. The error growth is similar on both the modelling and validation periods which is an important argument of validation. Model **M**_C_^*^ is non autonomous, consequently its synchronization on the observed dynamics is ensured by the external forcing *T*^obs^(*t*) and *W*^obs^(*t*). Therefore, it is possible to apply a direct comparison between the observed time series and the modelled CO_2_ concentration (Fig. 4, first row). No data set was found available to validate the model **M**_C_^***^ on an independent period, in particular the high values observed during the 1970s, when the number of visitors was particularly high.

### Models characterization

Various approaches can be used to detect and characterize chaotic dynamics. Chaos requires two main conditions: determinism and unpredictability. If determinism can be considered as a reasonable hypothesis in many situations, it should be proved robustly when trying to detect chaos from observational time series, since determinism is an essential condition of chaos^42^. Few approaches have been developed for this purpose, for example, the Direct Test for Determinism^67^ and the global modelling technique^27^ used in the present study. Unpredictability results from the existence of chaotic attractors in the phase space, that is, a bounded trajectory that will—strictly speaking—neither loop back to itself (since it should be non periodic), nor present any crossing (to guarantee determinism). As a consequence, to detect chaos, the integrability of any system should be checked on a sufficiently long period to claim for chaos^46^. A chaotic attractor will also be characterized by specific properties: (1) a fractal geometry^68^, (2) the exponential divergence of the flow^69^, and (3) topological structure characterized by folding^45^. Specific methodologies have been developed to capture each of these different properties from observational time series. These methodologies are specific and cannot check for determinism at the same time. As a consequence, their ability to detect chaos can mainly be used when starting from deterministic equations. It is one important advantage of the global modelling technique: by extracting equations directly from observational time series, it enables, not only to detect determinism, but also to apply other characterization techniques to the obtained equations. In this way, finally, all the properties of chaos (if so) can be gathered in a consistent framework: deterministic equations, chaotic attractor reconstructed from these equations and characterized by a fractal dimension, a diverging flow, and a folded topological structure. In the present study, the GPoM algorithm^65^, was used to extract the equations, which can then be used to estimate locally reliable Lyapunov exponents^44^ and to characterize the divergence of the flow. The Kaplan-Yorke dimension *D*_KY_^43^ was used to estimate the dimension of the obtained attractors, and first return maps were used to detect foldings^70^.

### Supplementary Information

Supplementary Information.

## Data Availability

All the time series used to generate the scenarios are provided in Data S1. The use of the time series gathered in the cave is restricted, but the chaotic attractors obtained from them can be reproduced based on the models description provided in Supplementary Materials. The algorithms of the GPoM package were used to obtain these models; these are Open Source and directly downloadable on the *Comprehensive R Archive Network* at the following link: https://CRAN.R-project.org/package=GPoM.
