# Supplementary material for: Scenarios for the Altamira cave CO2 concentration from 1950 to 2100
Source: Sci Rep. 2024 May 6;14:10359. doi: 10.1038/s41598-024-60149-9 (PMC11074292; doi:10.1038/s41598-024-60149-9)
Supplement: Supplementary file 1 — Supplementary Information. [file 41598_2024_60149_MOESM1_ESM.pdf]

Supplementary Information for

Scenarios for the Altamira cave CO_2 concentration from 1950 to 2100

Marina Sáez, David Benavente, Soledad Cuezva, Mireille Huc, Ángel Fernández-Cortés, Arnaud Mialon, Yann Kerr, Sergio Sánchez-Moral, Sylvain Mangiarotti

To whom correspondence should be addressed: sylvain.mangiarotti@ird.fr (S.M.), scuezva@csic.es (S.C.)

Supplementary Note 1 – Study site

The Altamira cave (43º22’40’’N; 4º7’6’’W) is located in Cantabria. Declared a World Heritage Site by the UNESCO for the uniqueness of its prehistoric paintings and engravings, it belongs to the Altamira Cave Complex and Palaeolithic Cave Art of Northern Spain. The cave is located in the vadose zone (*i.e.* the unsaturated zone between the phreatic zone and the land surface) of a senile karstic system hydrologically unconnected. It is placed on the top of a hill at 158 m.a.s.l. with low slope gradients, an unstable and poorly defined drainage network with no evidence of significant groundwater circulation. The Saja River, more than 100 m far from and well below the cave, is the only permanent stream around and represents the general water table [71]. A heterogeneous soil covers the rocky lapiaz. This soil, with little profile development (30-70 cm thick), is poorly differentiated and includes artificial backfill, and has a total porosity of 48% with organic matter content around 10% [21]. A grassland vegetation cover (C3 plants) develops from this soil. The area has an oceanic climate, specifically warm temperate *Cfb* type in the Köppen-Geiger classification, with temperate summer and no dry season. The annual mean temperature and total precipitations are around 14 ºC and 1400 mm, respectively [10].

Supplementary Note 2 – data description and preprocessing

1. Data

The detailed description of the observational time series used in the study is provided in the following paragraphs. Their main characteristics are summarized in Supplementary Table 1.

1.1 *In situ* data

Several *in situ* data sets were considered in the study, including variables measured inside (CO_2 concentration) and outside the cave (air temperature and volumetric water content in the soil). The positions of the sensors are shown in Fig. 1B. The original time series used in the analysis are shown in Supplementary Fig. 1, as well as the effect of their preprocessing. Depending on the context, the time series were used for modelling and/or validation. Their detailed description is given hereafter.

A micro-environmental station recorded the CO_2 concentration and the microclimatic data [72] inside the Polychromes Hall. It consisted of an 8-channel, 16-bit datalogger (COMBILOG TF 1020, Theodor Fiedrich & Co., Germany) with a suite of probes for CO_2 concentration (ITR 498 ADOS, Germany). The time series of CO_2 concentration (in ppm) was measured in the Polychromes Hall air during the period 28 March 2007 to 27 September 2012. Different sampling time were used since 2007 until 28 April 2009 (5 minutes), until 01 October 2009 (10 minutes), until 25 November 2009 (30 minutes), and until 24 November 2010 (60 minutes) and for the rest of the time series (15 minutes).

Complementary data sets of CO_2 concentration were also used, mainly for validation. From 23 July 2004 to 13 September 2005, records of CO_2 concentration were performed with a 15-minute sampling in the Polychromes hall [19-20]. Still in the same hall, records of CO_2 concentration were performed with a 60-minute sampling from 31 January 1997 to 07 August 1998 [15].

A weather station located on the hill above the cave, with two autonomous dataloggers, stored the air temperature (HOBO U23 Pro v2, Onset, Bourne, MA, USA), and the soil VWC at 5 cm depth (HOBO U12, Onset, Bourne, MA, USA, equipped with an ECHO EC-10 of Decagon Devices). The time series of the outside temperature (in Celsius) was measured during the period 28 March 2007 to 27 September 2012 (15 minutes sampling time). The time series of the Volumetric Water Content (VWC) in the upper layer of soil lying on the surface over the cave was measured during the period 20 June 2007 to 27 September 2012. This variable is expressed as fraction of the total volume (in m^3/m^3), therefore it cannot surpass the soil porosity, which is, according to laboratory characterizations, 0.48 m^3/m^3 [21]. The sampling time was 30 minutes until 03 February 2010, 60 minutes until 24 November 2010 and 30 minutes for the rest of the time series.

In general, the observed time series are characterized by a short time scale superimposed to the seasonal time scale signal. A low pass filter was applied to the time series of CO_2 concentration, the outside temperature and the VWC, since this study is focused on modelling the seasonal scale. The time series were smoothed using a local weighted regression [73] with a smoothing parameter α of 0.1 in all cases (except the CO_2 time series of 1997-98, for which the smoothing parameter was 0.25). The smoothed time series were re-sampled to daily values by averaging the values. The gaps in the resulting time series were moderate in size, therefore they were filled using cubic spline interpolation following previous analyses using the global

modelling technique [33]. However, the time series of CO_2 of 2007-2012 had three gaps longer than two months and smaller gaps as well. Only the small gaps of this time series were filled, whereas the long ones were kept as they were initially (see Supplementary Fig.1C).

The time series of the soil VWC shows an inter-annual decrease as a consequence of an artificial drift, which is commonly observed with the type of probe used to collect the data, and needs to be corrected [74]. To estimate and remove the drift, a moving average was calculated from 2009 to 2011 with a one-year sliding window and the result was smoothed using the local weighted regression. When the drift starts, its value must be zero; therefore, we subtracted from the filtered moving average its first value. Finally, the estimated drift was subtracted from the VWC time series.

1.2 Soil Moisture – The SMOS satellite data

SMOS satellite is the first mission dedicated to the measurements of surface soil moisture from space. Initiated by the end of the 1990s, it was launched on 02 November 2009, and started to collect scientific data of soil moisture on 17 January 2010 [50]. As the first months were dedicated to the commissioning phase, data are available since May 2010 until now.

Starting from the SMOS level 2 product, a specific processing, more adapted than the SMOS level 3 product commonly used, was considered in order to take the location of the site of study (~4.5 km from the ocean) and the large footprint of the SMOS measurements [75] into account. This processing enabled to keep the maximum of valid measurements in the crossing zone between ocean and continent at the closer vicinity to the studied site. This product was generated on a 15 km resolution grid and corresponds to version 700. Three time series were generated. These were compared to the *in situ* time series. Only the one enabling the best agreement with the *in situ* time series was kept. The selected time series is shown in Supplementary Fig. 4 and its area of selection on Fig. 1D.

The following processing was then applied to the time series. Initially irregular, the sampling time was homogenized to one value every 12 hours. Note that, as for any other forcing time series, its sampling time is double than that of the CO_2 time series because it is required by the Runge-Kutta method, which was chosen for numerical integration. In the first place, the original time series were interpolated to one value per second by means of the Stineman's interpolation [76]. Then the values were aggregated using the median on 12-hour windows: 14:00-01:59 and 02:00-13.59, centered at 8.00 and 20:00 (because Altamira *in situ* time series are centered at 20:00 too). The resulting time series were smoothed using a LOESS smoothing with parameter α equal to 0.05. Because the means of the satellite and the *in situ* time series were quite different (0.251 and 0.367 m^3/m^3, respectively), the satellite time series were re-calibrated. First, the mean of the SMOS time series was modified to match the mean of the *in situ* time series by adding a constant (0.115846). Then, a dumping function was used on the corrected SMOS time series to smooth excessively large maxima. The R^2 coefficient between the *in situ* and the preprocessed satellite data was of 78%. The time series at the closest vicinity to the site of study was also the one in with best agreement with the *in situ* observations. Only this one was considered for the analyses.

1.3 Soil Moisture – The ESA CCI SM product

The *European Space Agency*'s *Climate Change Initiative for Soil Moisture* (ESA CCI SM) has developed a merging algorithm to generate long-term (1978-2020) data for soil moisture [52].

The ESA CCI SM v4 product was used in the present study. This data set is exclusively based on satellite data. It merges active, passive, and combined active-passive products. These are sampled to daily images on a regular grid of 0.25° space resolution, at global scale. Six grid points were tested in the study. The quality of the extracted time series highly depends on the availability of the original data sets at the site of study. At the closest grid point here considered, data were particularly scarce up to 2012, therefore the next closest grid point was used instead (see Fig. 1D), from September 2003 (to 30 December 2012).

This time series was then re-sampled at a 12-hour sampling time using the Stinemann's interpolation. Due to important differences of means in comparison to the *in situ* time series, a recalibration was applied (see Supplementary Fig. 5 methods Section).

1.4 Soil Moisture – The ESM Global Multi-layer Soil Moisture Products

Another product, the *Earth System Model* (ESM) Global Multi-layer Soil Moisture Product based on the combination of (1) satellite observations, (2) re-analyses and (3) outputs of land surface models, was used in the study. This is a long-term, gap-free, multilayer soil moisture product on the period 01 January 1970 to 31 December 2016 [51].

This data set provides a gridded product of 0.5° resolution with a daily sampling time. Time series of soil moisture (0-10 cm depth) were extracted at the closest grid points (see Fig. 1D). The two time series on the coastal area were directly rejected as inconsistent and a single time series was kept for the analysis (see Supplementary Fig. 6).

The selected time series was first re-sampled at a 12-hour sampling time using cubic splines. As for the other data sets, the means of this time series and the *in situ* time series being significantly different (0.255 and 0.367 m^3/m^3, respectively), the time series was recalibrated following the procedure described in methods Section.

1.5 Land Surface Temperature – The MODIS data set

The brightness temperature measured from satellite in the optic bands can be used to estimate land surface temperature.

The longest satellite data set presently available for Land Surface Temperature is the MODIS11-C3 v6 product, provided by the *National Aeronautics and Space Administration* (NASA) with a monthly sampling time on a 0.05° grid, and available from 15 February 2000 to 15 June 2021. The time series (see Supplementary Fig. 7) located at the closest vicinity to the cave of Altamira was extracted (see Fig. 1D).

This time series was up-sampled to one value every 12 hours, by means of cubic splines. As for soil moisture time series, the sampling time was doubled in comparison to the CO_2 time series (12-hour vs. 24-hour) as required by the Runge-Kutta method. To ensure the consistency with the processing applied to the other variables, a LOESS smoothing [73] was then applied to the up-sampled time series, with a smoothing parameter of 0.05. A recalibration, whose description is provided in methods Section, was also applied.

1.6 Land Surface Temperature – The ECMWF model

For more remote periods (no appropriate LST data from satellite was available before 2000), re-analyses from the *European Centre for Medium-range Weather Forecasts* (ECMWF) were used.

The ERA5 product was chosen. This is a gridded product of hourly sampling time and 0.1° space resolution available from 1950 to 2020. The grid pixel considered in the study is located at (43.15°N, 355.85°E) as shown in Fig. 1D.

A time series of one measurement every 12h (at 8:00 and 20:00) was retrieved and smoothed with smoothing parameter 0.008 for temperature and 0.006 for VWC (Supplementary Figs. 8 and 9). A recalibration, whose description is provided in methods Section, was also applied.

1.7 The IPCC climatic scenarios

For running scenarios up to the end of the 21st century, simulations of the *Intergouvernmental Panel on Climate Change* (IPCC) were used. These simulations are based on a General Circulation Model of the *European Centre for Medium-Range Weather Forecasts*. Its detailed description is given in the Special Report on the Emissions Scenarios (SRES) published by the *Intergouvernmental Panel on Climate Change* (IPCC) in 2000 [77]. The considered scenarios were all run from 1990 based on observed conditions originally gathered for the time period 1860-1990 [55]. Originally run with a 24-minute time step [78], outputs are provided as a gridded product of monthly sampling time. To run model $\mathbf{M}_C^{**}$, four IPCC scenarios were considered:

The ECHAM4 A2 assumes a very heterogeneous world, largely based on self-reliance, with a continuation of the local governing modes. It is characterized by a continuous increasing of the populations associated with slow economic growth and slow technological changes. Time series of surface temperature and soil moisture simulated under this scenario are presented in Supplementary Figs. 10 and 11. At the site of study, considering a sliding window of 5 years, it is estimated that the increase of temperature reaches +3.5°C by 2100. The behaviour is however much more complex since it exhibits both higher maxima and lower minima. The grid point the closest to Altamira coordinates, located at (44,269°N, 355,718°E) was used (see Fig. 1A). It has latitudinal and longitudinal resolutions of 2.8° and 2.8°, respectively.

The ECHAM4 B2 scenario assumes a lower rate of population growth (in comparison to A2) in a world committed to local solutions for economic, social and environmental sustainability. It is estimated by the corresponding simulation that this scenario, now considered as unreachable, would have enabled a moderate increase of temperature on average (+2.5°C by 2100) with relatively moderate evolutions in the distribution of extreme values (see Supplementary Figs. 12 and 13). The grid point is the same as for scenario A2.

The ECHAM4 A1B assumes the world population will peak at mid-century and then, it will decrease. It assumes also quick economic growth with rapid introduction of more efficient new technologies and balanced use of multiple energy sources. The grid point is the same as for scenarios A2 and B2. This scenario would lead to an increase of temperature of +3°C on average by 2100, with no significant changes in the distribution of extreme values (see Supplementary Figs. 14 and 15).

The ECHAM4 B1 assumes the same population evolution as A1B. Scenario B1 assumes that clean and efficient technologies will be introduced as well as global solutions leading to sustainability and equity. The grid point is located on the Iberian peninsula and characterized by a very low resolution: 5.6° and 3.2° latitudinal and longitudinal resolutions, respectively. This scenario would lead to a very moderate increase of temperature on average (+2°C by 2100) with no significant changes in the distribution of extreme values (see Supplementary Figs. 16 and 17).

1.8 The number of visitors

The number of visitors has strongly varied from the early 1950s to the present. Annual data from 1952 were provided by the *Museum of Altamira* (Supplementary Table 11). Six main periods can be distinguished: an increasing number of visitors from moderately high in 1952 to extremely high in 1974, a quick decrease from 1975 to 1977, no visit from 1978 to 1981, a low controlled level of visits from 1982 to 2002, no visits from 2003 to 2013 and an even lower number of controlled visits from 2013 to the present. The schedule of the visits from 1952 to 1977 is unknown. In the period 1982-2002 there were five groups in the morning, consisting each one in 5 tourists plus a guide; the cave was closed on Mondays. From 2014, there is a single group of 5 tourists plus 2 guides every Friday.

2. *Time series recalibration*

2.1 Soil moisture products recalibration

Notable differences in the statistical characteristics (means and variance) are obviously observed between the considered products. This is expected since *(i)* they have very different spatial resolution (from very local for *in situ* data to very large resolution for the combined products); *(ii)* their construction is based on different physical (the response to a local electromagnetic field for *in situ* measurements, the natural emission of passive microwave for SMOS, etc.) and statistical processes (a combination of heterogeneous data sets for ESA CCI SM scaled using the model GLDAS, and ESM products); and *(iii)* the estimates are observed/reconstructed at different soil depths.

To make the comparison possible between these different data sets, it was chosen to recalibrate the satellite and hybrid time series, taking the *in situ* measurements as a reference. The *in situ* time series presenting a short overlap with some data sets, the following strategy was chosen: First, the data set presenting the longer time span (the ESM product) was calibrated on the *in situ* time series. Then, the other products (CCI, ECHAM4) were calibrated either on the calibrated ESM time series or on the in situ time series (in each case, the best fit was chosen). In all the cases, a linear fitting was applied enabling to calibrate at the same time the means and the variance of the time series. The data for the IPCC scenarios A1B and B1 present some calibration problems (too high p-value for the intercept and poor r-square, respectively) and, as a consequence, they cannot be considered as realiable as the A2 and B2 scenarios. The results of this inter-calibration are summarized in Supplementary Table 2.

2.2 Temperature products recalibration

The temperature products do also present significantly different statistical means and variances in comparison to the *in situ* records. Here also, such differences are expected due to very different spatial resolution (from very local for *in situ* data to large and very large for satellite and models resolution), and methodologies (direct versus indirect measurements or physical modelling). The overlapping of the *in situ* recording with the three other data sets is complete, therefore it was used as direct reference in all cases. The results of the calibration of the temperature time series are summarized in Supplementary Table 3.

Supplementary Note 3 – Obtained Models

Model $\mathbf{U}_C$:

The model $\mathbf{U}_C$ obtained for the concentration of CO_2 from a single time series $C^{obs}(t)$ is based on a canonical structure (Eqs. 5). It is composed of N_p = 13 terms, including 11 ones in the polynomial

$$\begin{aligned} P_C = &\alpha_0 C_2^2 - \alpha_1 C_2^3 + \alpha_2 C + \alpha_3 C C_2 C_3 + \alpha_4 C C_2^2 - \alpha_5 C^2 - \alpha_6 C^2 C_3 \\ &- \alpha_7 C^2 C_2^2 + \alpha_8 C^3 - \alpha_9 C^3 C_2 - \alpha_{10} C^4 \end{aligned} \quad \text{(S1)}$$

of which parameter values are provided in Supplementary Table 5. The model enables a consistent reconstruction of the phase space, both visually, and in terms of range (see Fig. 2). Error growth revealed very comparable results on both the modelling and independent windows, providing a robust argument of validation (Supplementary Table 8). This dynamic is characterized by a relatively low predictability (horizon of predictability H_P of ~30 days for a maximum error of 5% with a 90% confidence level, see Supplementary Fig. 3A). This low predictability is very likely due to the multiple external influences acting on the cave atmosphere dynamics onto which the cave has weak dynamical feedback only.

The obtained model exhibits all the properties of chaos gathered in a consistent object. Determinism – which is a necessary condition for chaos that has to be demonstrated when starting from observational time series [42] – is ensured by the equations of ordinary differential structure. All the other properties of chaos (see Supplementary Table 4) are also retrieved in the attractor obtained from these equations: positive first Lyapunov exponent (λ_1 = +8.8·10^{-5} ±1.2·10^{-5}), fractal dimension (D_{KY} = 2.09 ±0.02), and presence of folding in the model flow, whose first return map is characterized by a multi-modal structure containing at least four monotonic branches (see Fig. 2C).

Model $\mathbf{U}_T$:

The model $\mathbf{U}_T$ here obtained for the outside temperature from a single time series $T^{obs}(t)$ is also based on a canonical structure (Eqs. 5). It is composed of N_p = 30 terms, including 28 ones in the polynomial

$$\begin{aligned} P_T = &\mu_0 - \mu_1 T_3 + \mu_2 T_3^2 - \mu_3 T_3^3 + \mu_4 T_3^4 - \mu_5 T_3^5 - \mu_6 T + \mu_7 T T_3 - \mu_8 T\, T_3^2 \\ &+ \mu_9 T\, T_3^3 - \mu_{10} T\, T_3^4 - \mu_{11} T\, T_2 + \mu_{12} T\, T_2 T_3 + \mu_{13} T_1^2 - \mu_{14} T_1^2 T_3 \\ &+ \mu_{15} T_1^2 T_3^2 - \mu_{16} T_1^2 T_3^3 + \mu_{17} T_1^2 T_2 - \mu_{18} T_1^2 T_2 T_3 - \mu_{19} T_1^3 \\ &+ \mu_{20} T_1^3 T_3 - \mu_{21} T_1^3 T_3^2 - \mu_{22} T_1^3 T_2 + \mu_{23} T_1^3 T_2 T_3 + \mu_{24} T_1^4 \\ &- \mu_{25} T_1^4 T_3 + \mu_{26} T_1^4 T_2 - \mu_{27} T_1^5 \end{aligned} \quad \text{(S2)}$$

of which parameter values are provided in Supplementary Table 6. The model enables a consistent reconstruction of the phase space, both visually, and in terms of range (Fig. 2). Here also, the error growth revealed comparable results on both the modelling and independent windows, providing a strong argument of validation. In comparison to the concentration of CO_2, this dynamic is characterized by excellent predictability at seasonal scale (horizon of predictability H_P of ~120 days for a maximum error of 5% with a 90% confidence level, see Supplementary Fig. 3B).

This model also exhibits all the properties of chaos (Supplementary Table 4), all gathered in a consistent object: determinism is ensured by the equations of ordinary differential structure, positive first Lyapunov exponent (λ_1 = +9.7·10^{-4} ±2.2·10^{-4}), fractal dimension (D_{KY} = 2.13 ±

0.03), and presence of folding in the model flow, characterized by a multi-modal structure of the first return map with three monotonic branches (see Fig. 2C).

Model $\mathbf{U}_W$:

The model $\mathbf{U}_W$ obtained here for the soil water content from a single time series $W^{obs}(t)$ is also based on a canonical structure (Eqs. 5). It is composed of N_p = 27 terms, including 25 ones in the polynomial

$$\begin{aligned} P_W = \tau_0 - \tau_1 W_3 + \tau_2 W_3^2 + \tau_3 W_2 - \tau_4 W_2 W_3 + \tau_5 W_2 W_3^2 - \tau_6 W_2^2 + \tau_7 W_2^3 - \tau_8 W \\ + \tau_9 W W_3 - \tau_{10} W W_3^2 - \tau_{11} W W_2 + \tau_{12} W W_2 W_3 - \tau_{13} W W_2 W_3^2 + \tau_{14} W W_2^2 \\ - \tau_{15} W W_2^3 + \tau_{16} W^2 - \tau_{17} W^2 W_3 + \tau_{18} W^2 W_2 - \tau_{19} W^2 W_2 W_3 - \tau_{20} W^2 W_2^2 \\ - \tau_{21} W^3 + \tau_{22} W^3 W_3 - \tau_{23} W^3 W_2 + \tau_{24} W^4 \end{aligned} \quad \text{(S3)}$$

of which parameter values are provided in Supplementary Table 8. The model enables a consistent reconstruction of the phase space, both visually, and in terms of range (Fig. 2). Here again, the error growth revealed comparable results on both the modelling and independent window, providing a strong argument for validation. As for the external temperature, its dynamics is characterized by an excellent predictability at seasonal scale (horizon of predictability H_P of ~110 days for a maximum error of 5% with a 90% confidence level, see Supplementary Fig. 3C).

Here again, the model exhibits all the properties of chaos (Supplementary Table 4) gathered in a consistent object: determinism is ensured by the equations of ordinary differential structure, first Lyapunov exponent is positive ($\lambda_1 = +1.4 \cdot 10^{-3} \pm 1.0 \cdot 10^{-4}$), fractal dimension ($D_{KY} = 2.39 \pm 0.02$), and presence of folding in the model flow, characterized by a multi-modal structure of the first return map with several monotonic branches (see Fig. 2C).

Model $\mathbf{M}_{CTW}$:

This multivariate model $\mathbf{M}_{CTW}$ (Eqs. 15-20) is six-dimensional since it involves C the concentration of CO_2 in the cave, T, $\dot{T}$ ($=T_2$) and $\ddot{T}$ ($=T_3$) the external temperature and its two first derivatives, and W, $\dot{W}$ ($=W_2$) the volumetric water content above the cave and its first derivative. Parameter values are provided in Supplementary Table 9. Eqs. (16-18) correspond to the univariate model $\mathbf{U}_T$ obtained for the outside temperature, already presented higher in the text (with polynomial U_T defined in Eq. S2). The other two polynomials are defined as

$$P_C(T, T_2, W) = \alpha_0 - \alpha_1 W + \alpha_2 W^2 - \alpha_3 T_2 - \alpha_4 T + \alpha_5 TW - \alpha_6 TW^2 + \alpha_7 T^2 - \alpha_8 T^2 W \quad \text{(S4)}$$

$$\begin{aligned} P_W(T, T_2, W) = -\gamma_0 + \gamma_1 W - \gamma_2 W^2 - \gamma_3 T_2 W + \gamma_4 T_2 W^2 + \gamma_5 T \\ - \gamma_6 TW + \gamma_7 TW^2 - \gamma_8 T^2 + \gamma_9 T^2 W + \gamma_{10} T^3 \end{aligned} \quad \text{(S5)}$$

Several projections of the model phase space are shown in Fig. 3B. The temperature forcing generates a chaotic behaviour (as demonstrated by the two-branch first return map Fig. 3C) on the concentration of CO_2 (and on the soil moisture also). The dynamics is moderately developed in comparison to the observations (maximum temperature values are in the range [17.4; 18.9] ºC for the model, versus [16.5; 18.5] ºC for the observations). Despite this, it shows that the temperature can, not only generate chaotic oscillations on the cave micro-atmosphere, but also synchronize them on its pseudo-periodic behaviour. Moreover, the complexity of the transient (not shown) revealed that temporarily perturbed conditions may give rise to more complexity on both the concentration of CO_2 and the soil moisture, with some temporary de-synchronization which is very consistent with the observed phase portrait.

The analysis of the error growth confirmed the low predictability of the concentration of CO_2 at seasonal scale (horizon of predictability H_P of ~10 days for a maximum error of 5% with a 90% confidence level, see Supplementary Fig. 3D) as already observed with the model $\mathbf{U}_C$.

The main properties of chaos were also retrieved (Supplementary Table 4): determinism is ensured by the equations of ordinary differential structure, and presence of folding in the model flow characterized by a multi-modal structure of the first return map with two monotonic branches (see Fig. 3C).

Models $\mathbf{M}_C^*$, $\mathbf{M}_C^{**}$ and $\mathbf{M}_C^{***}$:

The non autonomous model $\mathbf{M}_C^*$ (Eq. 2) was obtained by extracting the first equation of model $\mathbf{M}_{CTW}$ for the variations of the concentration of CO_2 (Eq. 15) and replacing the variables T and W by an explicit forcing $T^{obs}(t)$ and $W^{obs}(t)$ ensured in practice by genuine observational time series of external temperature and soil water content, either observed *in situ* with $T_{in\ situ}(t)$ and $W_{in\ situ}(t)$, from satellite with $T_{\mathrm{MODIS}}(t)$, $W_{\mathrm{SMOS}}(t)$ and $W_{\mathrm{CCI}}(t)$ or based on hybrid products with $T_{\mathrm{ECMWF}}(t)$, $T_{\mathrm{IPCC}}(t)$, $W_{\mathrm{ESM}}(t)$ and $W_{\mathrm{IPCC}}(t)$ (these are all synthesized in Supplementary Table 1). The model enables a very consistent reconstruction of the phase space, both visually, and in terms of range (Fig. 4B).

As described in the main text, additional forcings were added to the model M_C^* in order to account for the variations of the CO_2 level in the outside from 1950 to 2100 (model M_C^{**}, Eq. 3) and for the CO_2 introduced by the visitors' breath (model M_C^{***}, Eq. 4).

Supplementary Note 4 – Model ensemble size

For a general structure of univariate canonical form (Eqs. 5) involving one observational time series and its two first derivatives (that is for $n = 3$, e.g. T, $\dot{T}$ and $\ddot{T}$), the number of possible model is 10^{10} under quadratic ($q^{max} = 2$) and 10^{35} under cubic ($q^{max} = 3$) conditions. For a general structure of multivariate form (Eqs. 6) involving three observed variables without any derivatives (that is C, T and W), the number of models will be 10^{30} under quadratic, and 10^{145} under cubic conditions.

For general structures of multivariate form (Eqs. 6), the number of possible models can become tremendous. As an example, considering a set of variables including the observed variables and either their first derivative $(C, \dot{C}, T, \dot{T}, W, \dot{W})$, or their two first derivatives $(C, \dot{C}, \ddot{C}, T, \dot{T}, \ddot{T}, W, \dot{W}, \ddot{W})$, and allowing a maximal polynomial degree $q^{max} = 2$, will give rise, respectively, to $N_{mod} = 2^{84}$ and 2^{165} possible models. For a maximal polynomial degree $q^{max} = 3$, we will reach $N_{mod} = 2^{252}$ and 2^{660} possible models.

Supplementary Note 5 – Model adaptations

Model adaptations were applied in some cases. The first type simply consisted in reformulating an autonomous model into a non autonomous one. This can be done, basically, by removing some of the modelled variables and replacing them by the observed time series, used as an explicit forcing. This is what was done to obtain model $\mathbf{M}_C^*$ (Eq. 2) from $\mathbf{M}_{CTW}$ (Eqs. 15-20). However, under forcing conditions, and more specifically, due to the decreasing minimum value of the outside temperature, CO_2 concentrations lower than the outside concentrations were sometimes obtained. This was physically impossible and it directly results from the 5-year length of the original modelling window (2007-2012), long enough to catch the inter-annual dynamical behaviour, although too brief to detect the decadal or secular variations resulting from the increase of the CO_2 in the open atmosphere. To account for this effect, the algebraic term $+\Lambda_{Atm}$ $(C^{ext}(t) - C^{ext}_{2007})$ was added (see Eq. 3 obtained from Eq. 2) where $C^{ext}(t)$ denotes the CO_2 concentration in the outside atmosphere, C^{ext}_{2007} is its value on the January 2007, and $\Lambda_{Atm} = +0.20$ a coefficient in day^{-1} that was calibrated on the period 1990-2100 in order to satisfy the condition $C^{ext}(t) \leq C(t)\ \forall t$. This term was used in model $\mathbf{M}_C^{**}$ (Eq. 3) for all the long-term simulations. Observational time series from the *Mauna Loa Observatory* of the *Global Monitoring Laboratory* [79] were used for the period 1950-2022, linear extrapolations were used for the period 2022-2100. The time evolution $C^{ext}(t)$ being monotonous acts a positive trend in the future scenarios (limiting the minimum values of CO_2 reached by the corrected model) and as a negative trend in the past reconstruction. Finally, to account for the CO_2 exhalation by the visitors, a model $\mathbf{M}_C^{***}$ (Eq. 4) was adapted considering the number of visitors $V(t)$ as another forcing. To do so, the term $+\Lambda_{Vis}\, V(t)$ was also added with Λ_{Vis} the additional input of CO_2 to the cave atmosphere, per visitor and per day which was calibrated on the period 1997-1998. Since the models are run with a time step of 12h for the forcings and the only information available up to 1977 is the annual number of visitors, the daily number and distribution of visitors had to be reconstructed considering that there is a time with visitors input (8:00 to 20:00h) and a time without (20:00 to 8:00h). The timetable of the period 1982-2002 was projected backwards considering two different simulations (see Fig.6C-6D). For the first simulation, the number of visitors per year was divided homogeneously (except Mondays, when the cave is assumed to be closed). For the second simulation, more refined, the affluence of visitors was assumed to depend on the situation: maximal on Sundays, national holidays and the month of August; intermediate on Saturdays and the month of July and low the rest of the days (excluding Mondays).

Supplementary references

71. J. Elez, S. Cuezva, A. Fernandez-Cortes, E. Garcia-Anton, D. Benavente, J. C. Canaveras, S. Sanchez-Moral, A GIS-based methodology to quantitatively define an Adjacent Protected Area in a shallow karst cavity: the case of Altamira cave. *J Environ Manage* 118, 122-134 (2013).

72. S. Sanchez-Moral, S. Cuezva, A. Fernandez-Cortes, I. Janices, D. Benavente, J.C. Cañaveras, J. Elez, J.M. Gonzalez, V. Jurado, L. Laiz, M.C. Portillo, M.A. Rogerio, C. Saiz-Jimenez, Estudio integral del estado de conservación de la cueva de Altamira y su arte paleolítico (2007-2009). Perspectivas futuras de conservación. Monografías Nº24. Museo Nacional y Centro de Investigación de Altamira. pp. 1-397. Ministerio de Educación y Cultura. Secretaría General Técnica. Subdirección General de Documentación y Publicaciones (2014). Available on-line at: https://www.cultura.gob.es/dam/jcr:907536f2-b5f8-458a-b92a-37359b648699/pdf24-sanchez-moral-2014-conservacion-altamira.pdf

73. W.S. Cleveland, S.J. Devlin, Locally Weighted Regression: An Approach to Regression Analysis by Local Fitting. *Journal of the American Statistical Association* **83**(403), 596–610 (1988). https://doi.org/10.1080/01621459.1988.10478639

74. W.A. Dorigo, A. Xaver, M. Vreugdenhil, A. Gruber, A. Hegyiova, A. Sanchis-Dufau, D. Zamojski, C. Cordes, W. Wagner, M. Drusch, Global automated quality control of in situ soil moisture data from the International Soil Moisture Network. *Vadose Zone Journal* **12**(3), 1–21 (2013). https://doi.org/10.2136/vzj2012.0097

75. Y.H. Kerr, P. Waldteufel, P. Richaume, J.-P. Wigneron, P. Ferrazzoli, A. Mahmoodi, A. Al Bitar, F. Cabot, C. Gruhier, S. Juglea, D. Leroux, A. Mialon, S. Delwart, The SMOS Soil Moisture Retrieval Algorithm. *IEEE T. Geosci. Remote* **50**, 1384–1403 (2012). https://doi.org/10.1109/TGRS.2012.2184548

76. R.W. Stineman, A Consistently Well-Behaved Method for Interpolation. *Creative Computing* **6**, 54–57 (1980).

77. IPCC (2000). *Special report on emissions scenarios*, Cambridge University Press, Cambridge, UK.

78. E. Roeckner, K. Arpe, L. Bengtsson, S. Brinkop, L. Dümenil, M. Esch, E. Kirk, F. Lunkeit, M. Ponater, B. Rockel, R. Suasen, U. Schlese, S. Schubert, M. Windelband, *Simulation of the present-day climate with the ECHAM4 model: impact of model physics and resolution* Max-Planck Institute for Meteorology, Report No.93, Hamburg, Germany, 171pp. (1992).

79. P. Tans, K. Thoning, How we measure background CO_2 levels on Mauna Loa. NOAA Global Monitoring Laboratory, Boulder, Colorado (September 2020). https://gml.noaa.gov/ccgg/about/co2_measurements.html

80. Meehl, Gerald **(2003)**. *IPCC-DDC_NCARPCM_SRES_A1B: 120 YEARS MONTHLY MEANS National Center for Atmospheric Research USA*. World Data Center for Climate (WDCC) at DKRZ. https://hdl.handle.net/21.14106/45d60e83bb1b27f9b918b137d5024d9dfd177ab8

81. Gordon, Hal **(2005)**. *CSIRO_SRES_B1_SMC*. World Data Center for Climate (WDCC) at DKRZ. https://doi.org/10.1594/WDCC/CSIRO_SRES_B1_SMC

82. Stendel, Martin; Schmith, Torben; Roeckner, Erich; Cubasch, Ulrich **(2005)**. *EH4OPYC_SRES_A2_SMC*. World Data Center for Climate (WDCC) at DKRZ. https://doi.org/10.1594/WDCC/EH4OPYC_SRES_A2_SMC

83. Esch, Monika **(2005)**. *EH4OPYC_SRES_B2_SMC*. World Data Center for Climate (WDCC) at DKRZ. https://doi.org/10.1594/WDCC/EH4OPYC_SRES_B2_SMC

84. Gordon, Hal **(2005)**. *CSIRO_SRES_B1_TMP*. World Data Center for Climate (WDCC) at DKRZ. https://doi.org/10.1594/WDCC/CSIRO_SRES_B1_TMP

85. Stendel, Martin; Schmith, Torben; Roeckner, Erich; Cubasch, Ulrich **(2005)**. *EH4OPYC_SRES_A2_TMP*. World Data Center for Climate (WDCC) at DKRZ. https://doi.org/10.1594/WDCC/EH4OPYC_SRES_A2_TMP

86. Esch, Monika **(2005)**. *EH4OPYC_SRES_B2_TMP*. World Data Center for Climate (WDCC) at DKRZ. https://doi.org/10.1594/WDCC/EH4OPYC_SRES_B2_TMP

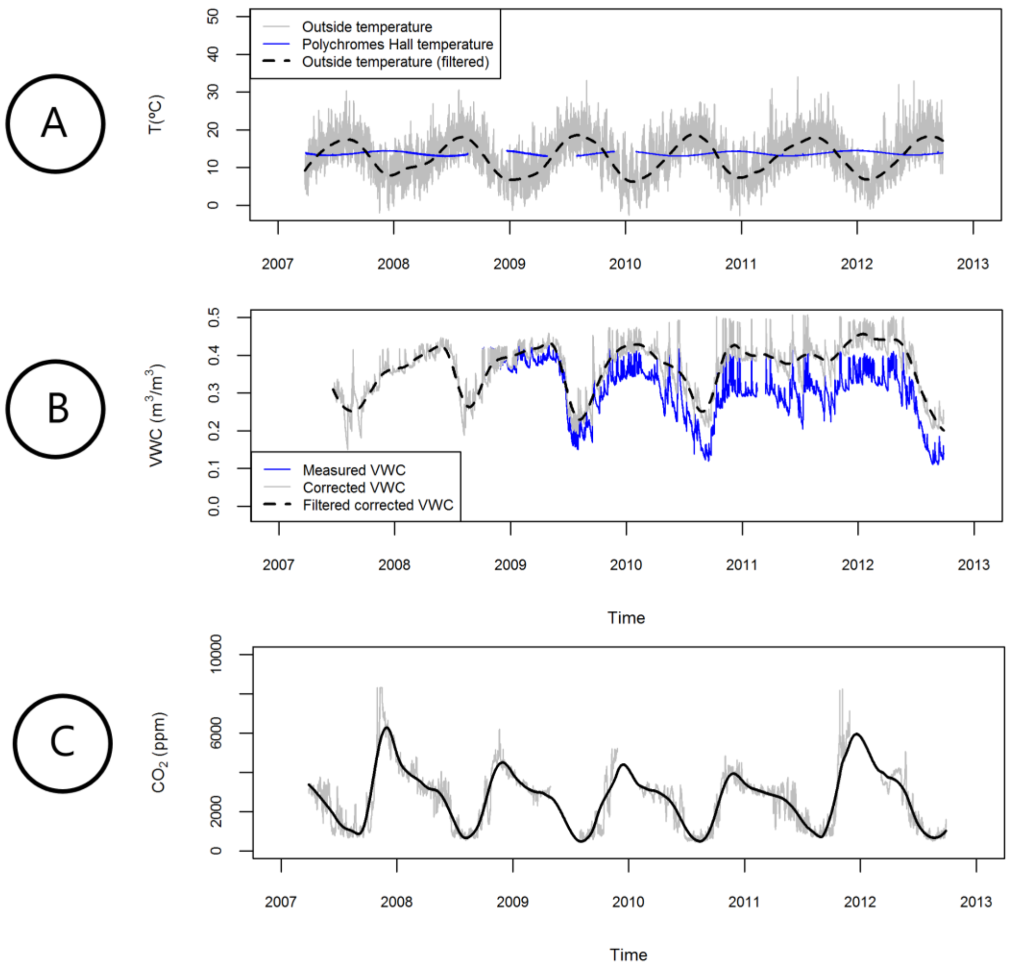

Fig. 1 ***In situ*** **time series.** Original (in gray) and pre-processed (in black) time series of the outside temperature (A) (temperature measured in the Polychrome hall is also provided in blue), of the volumetric water content in the soil at the vertical of the cave (B) (measurements before drift correction is provided in blue), and of CO_2 concentration observed *in situ* in the polychrome hall (C).

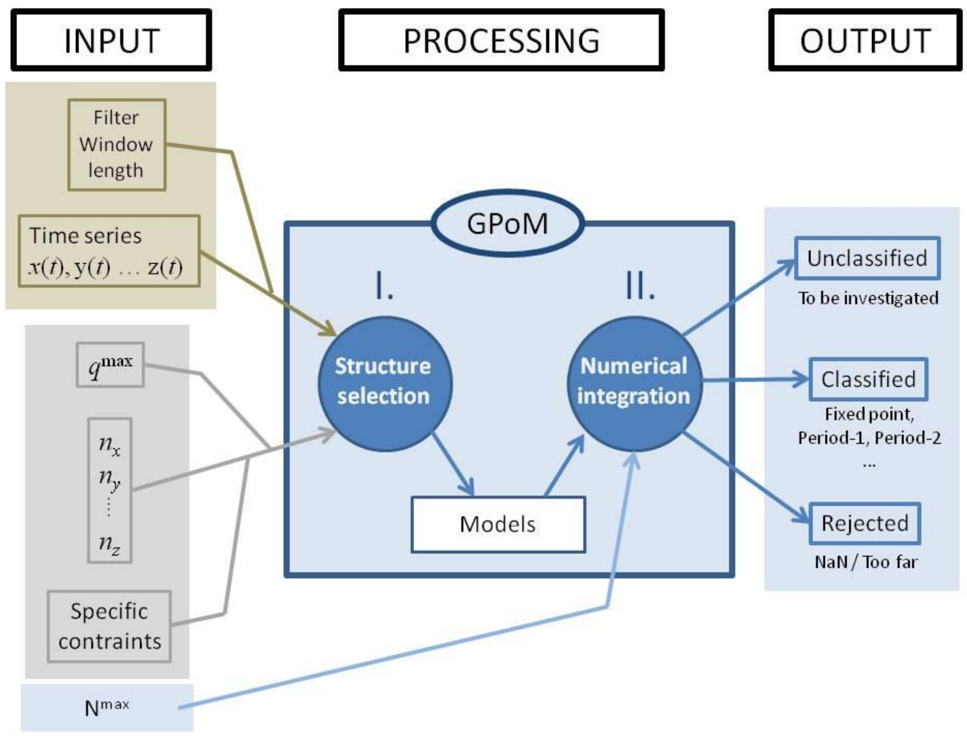

Fig. 2 General algorithm for obtaining dynamical equations from time series.

The Global Polynomial Modelling (GPoM) algorithm aims to obtain governing equations (or a good approximation of them) for a studied system, directly from observational time series. The algorithm requires a set of time series providing a proper description of the observed system and several parameters: the window length to be used to compute the derivatives of the input time series, the number of variable n_x, n_y, etc. to be derived from each time series (e.g. for $n_x = 3$, the original time $x(t)$ will be used together with its two first derivatives dx/dt and d^2/dt^2); the maximum polynomial degree q^{max} allowed in the numeric formulation, optionally some specific constraints to be applied to the equations (e.g. max number of parameters, forbidden couplings, etc.), and the maximum integration time steps N^{max}. In output, the models are separated into three groups: (i) rejected due to NaN or too far from the original data (>4·σ.); (ii) classified as fixed point, period-1, period-2, etc.; and (iii) unclassified (and thus requiring further analyses to be classified properly).

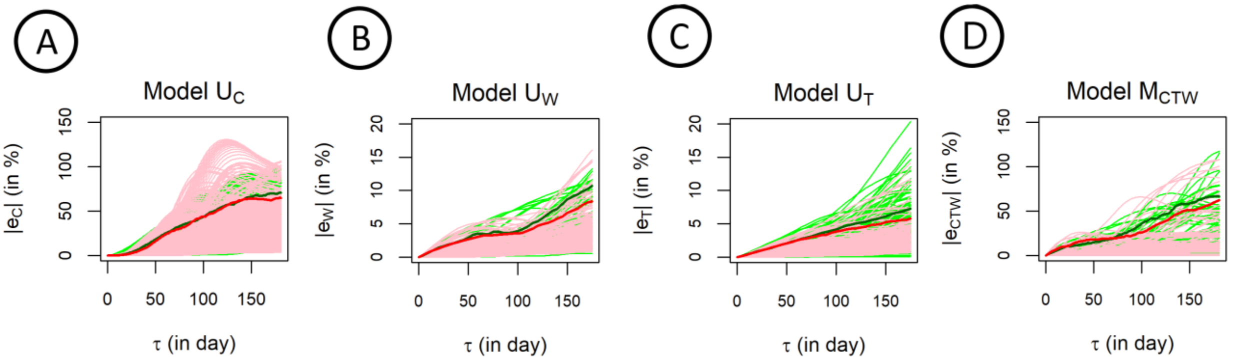

Fig. 3 Models error growth. The error growth of models $\mathbf{U}_C$, $\mathbf{U}_T$, $\mathbf{U}_W$ and $\mathbf{M}_{CTW}$ is shown in (A) to (D), respectively. For the multivariate model $\mathbf{M}_{CTW}$, only the error growth of variable C is provided since the dynamics of variables W and T strictly relies on models $\mathbf{U}_W$ and $\mathbf{U}_T$ for which validation is already provided in (A) to (C). All the particular simulations of the ensemble are shown in thin lines for both the modelling window (in pink) and the validation window (in green). The 90%-percentile is also provided for both ensembles, in thick lines (red and dark green, respectively).

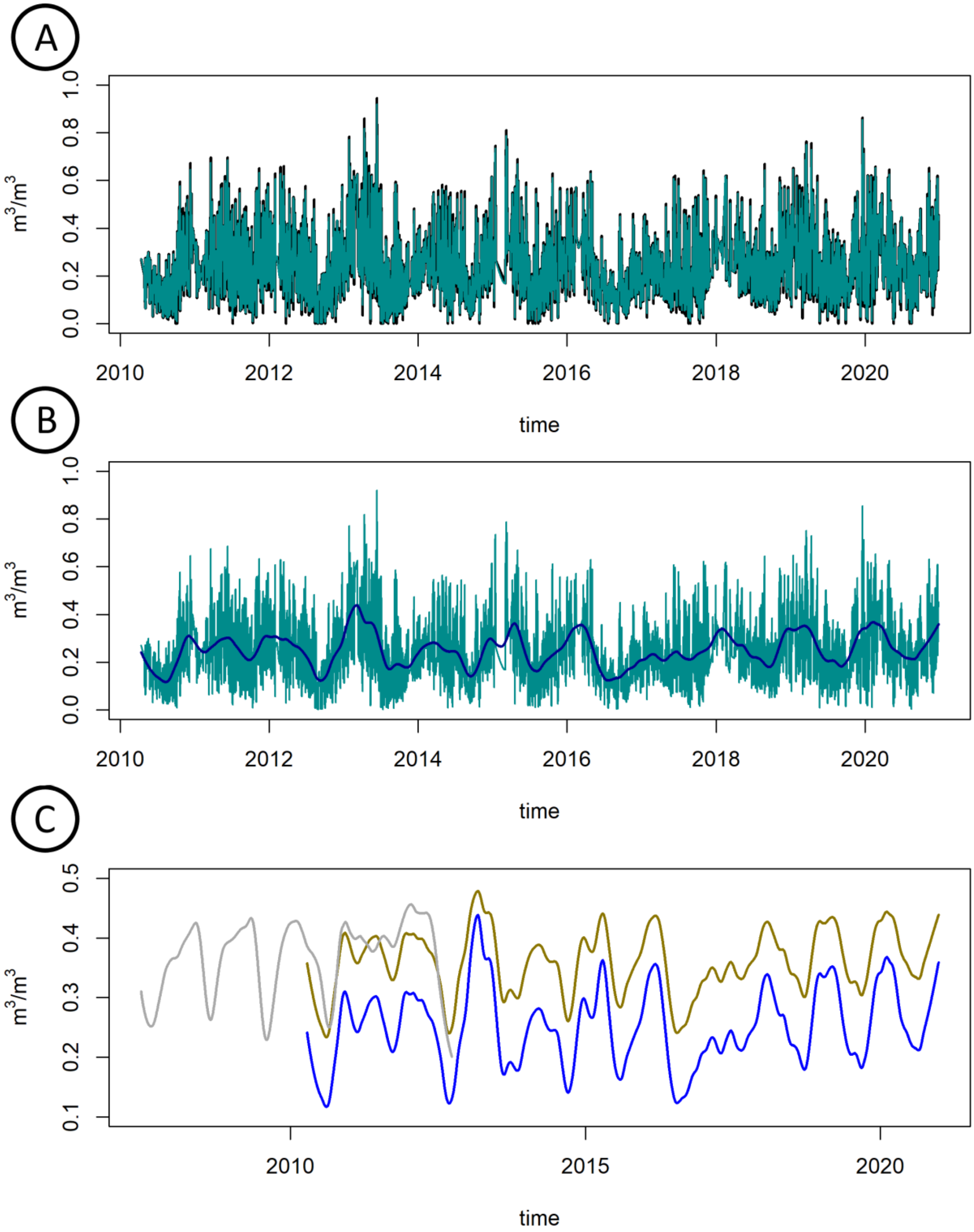

Fig. 4 Time series of volumetric water content from SMOS satellite. Original observations in black and re-sampled time series in blue (A); smoothed time series before calibration in dark blue (B), time series before and after calibration (blue and brown lines, respectively) where the *in situ* observations are also reported in grey (C). The calibration parameters are provided in Supplementary Table 2.

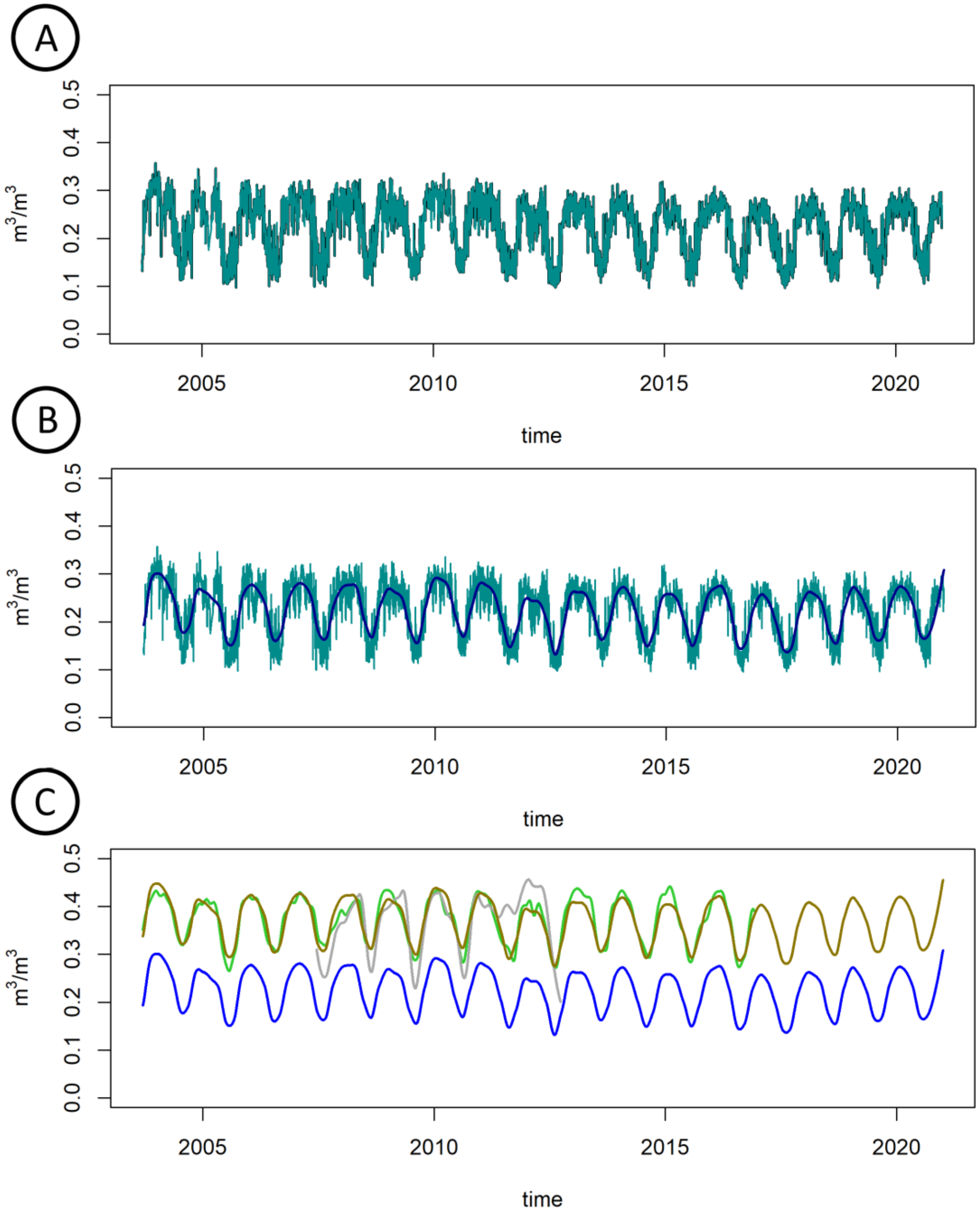

Fig. 5 Time series of volumetric water content from multisatellite product. Original observations of the ESA CCI SM product in black and re-sampled time series in blue (A); smoothed time series before calibration in dark blue (B), time series before and after calibration (blue and brown lines, respectively), where the *in situ* observations are also reported in grey and the ESM time series, used for the intercalibration, in green (C). The calibration parameters are provided in Supplementary Table 2.

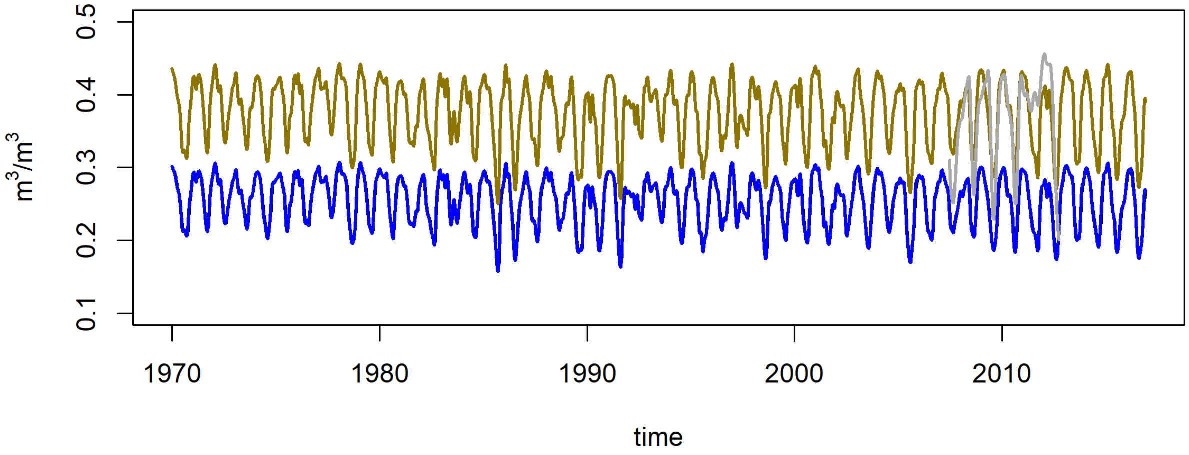

Fig. 6 Time series of volumetric water content from hybrid product. Original observations of the ESM Global Multi-layer Soil Moisture Product (blue line), after calibration (brown line), with the *in situ* observations also reported (grey line). The calibration parameters are provided in Supplementary Table 2.

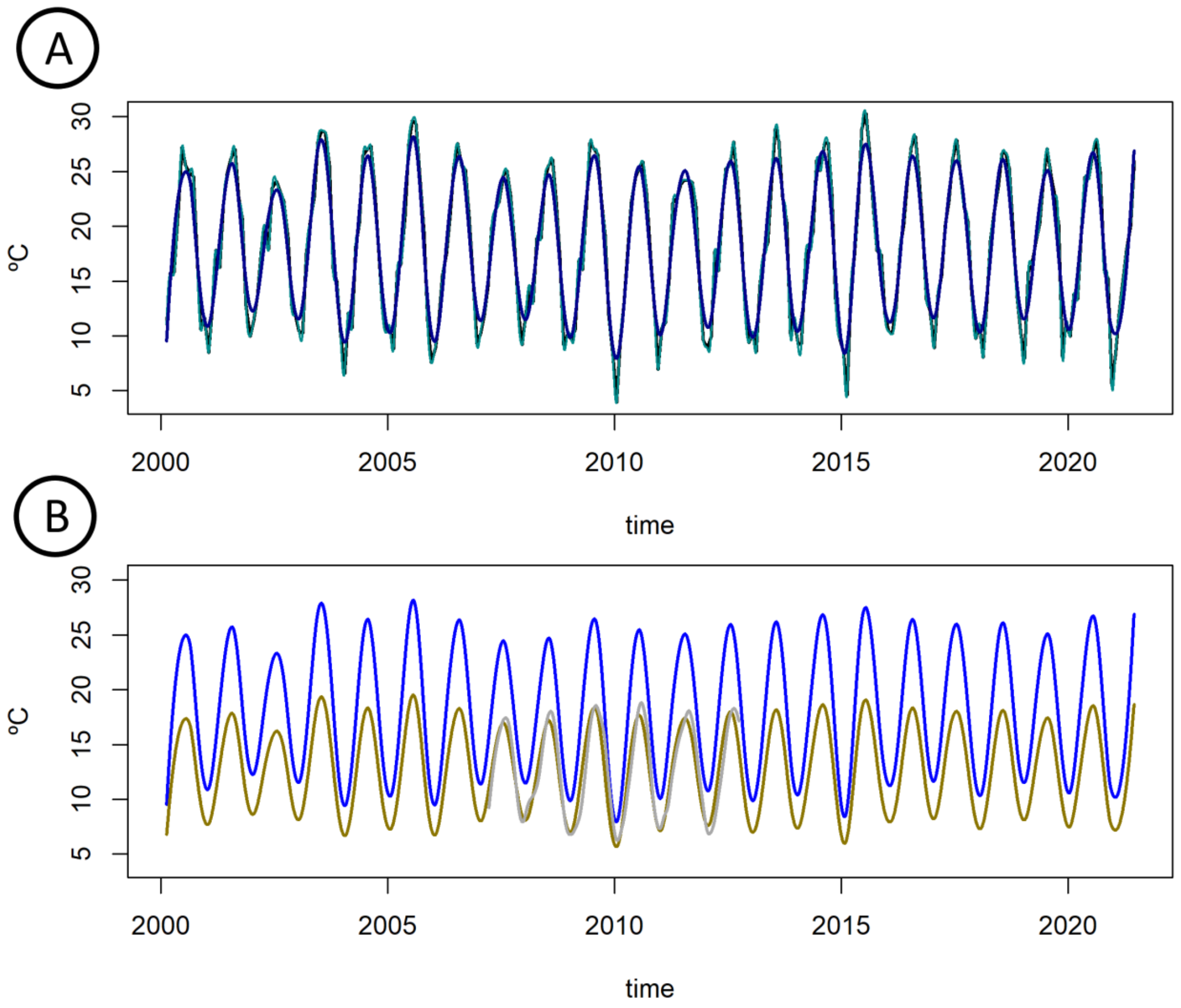

Fig. 7 LST Satellite time series. Original data (black), re-sampled data (light blue) and smooth data (dark blue) (A); smooth data before calibration (dark blue), after calibration (brown) and observational time series used for calibration (grey) (B). The calibration parameters are provided in Supplementary Table 3.

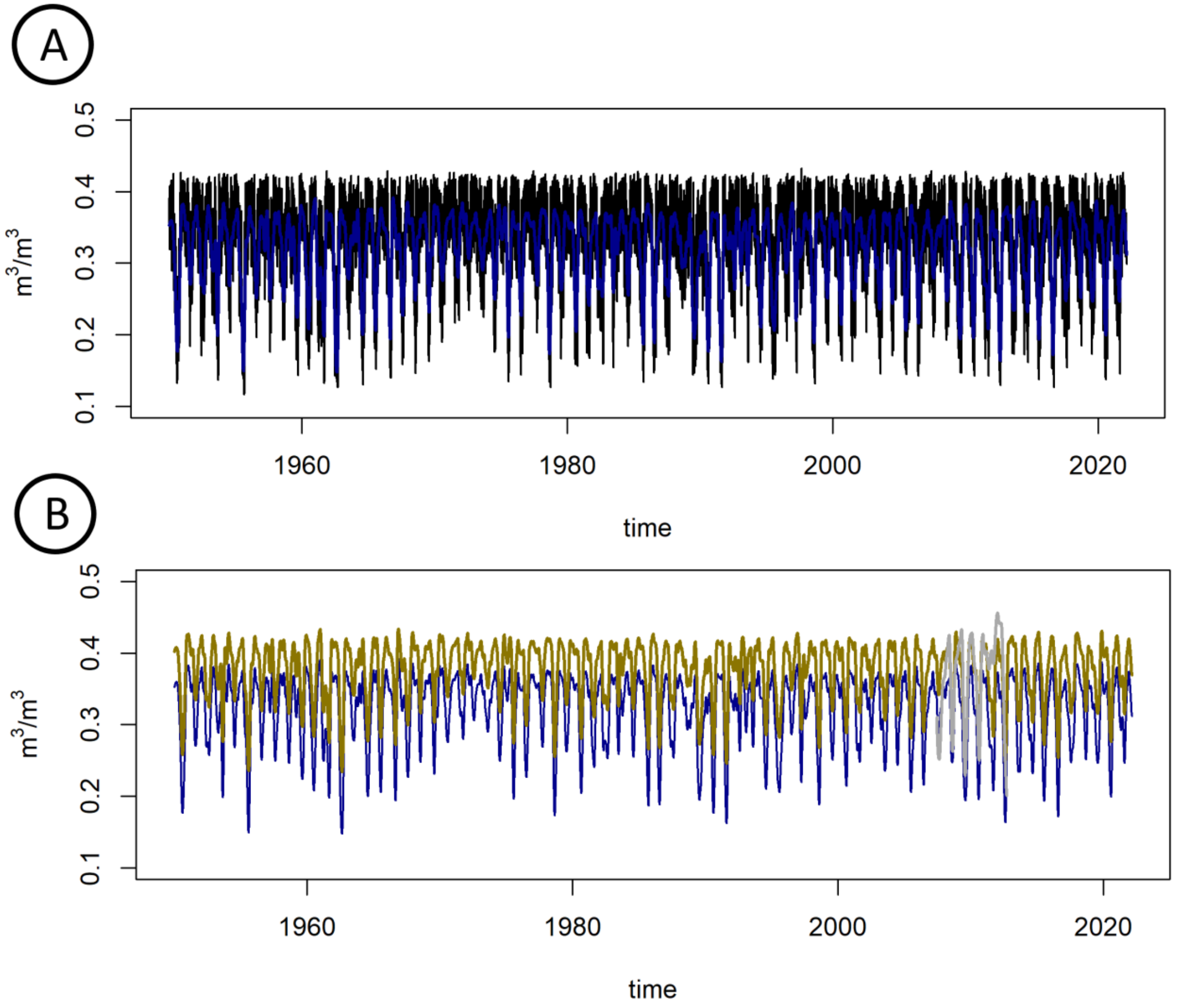

Fig. 8 Time series of volumetric water content from the ECMWF model. Original product of the ERA5 simulations of the ECMWF model, before and after smoothing (A); time series before and after calibration (in dark blue and brown, respectively) with the *in situ* observations also reported in grey (B). The calibration parameters are provided in Supplementary Table 2.

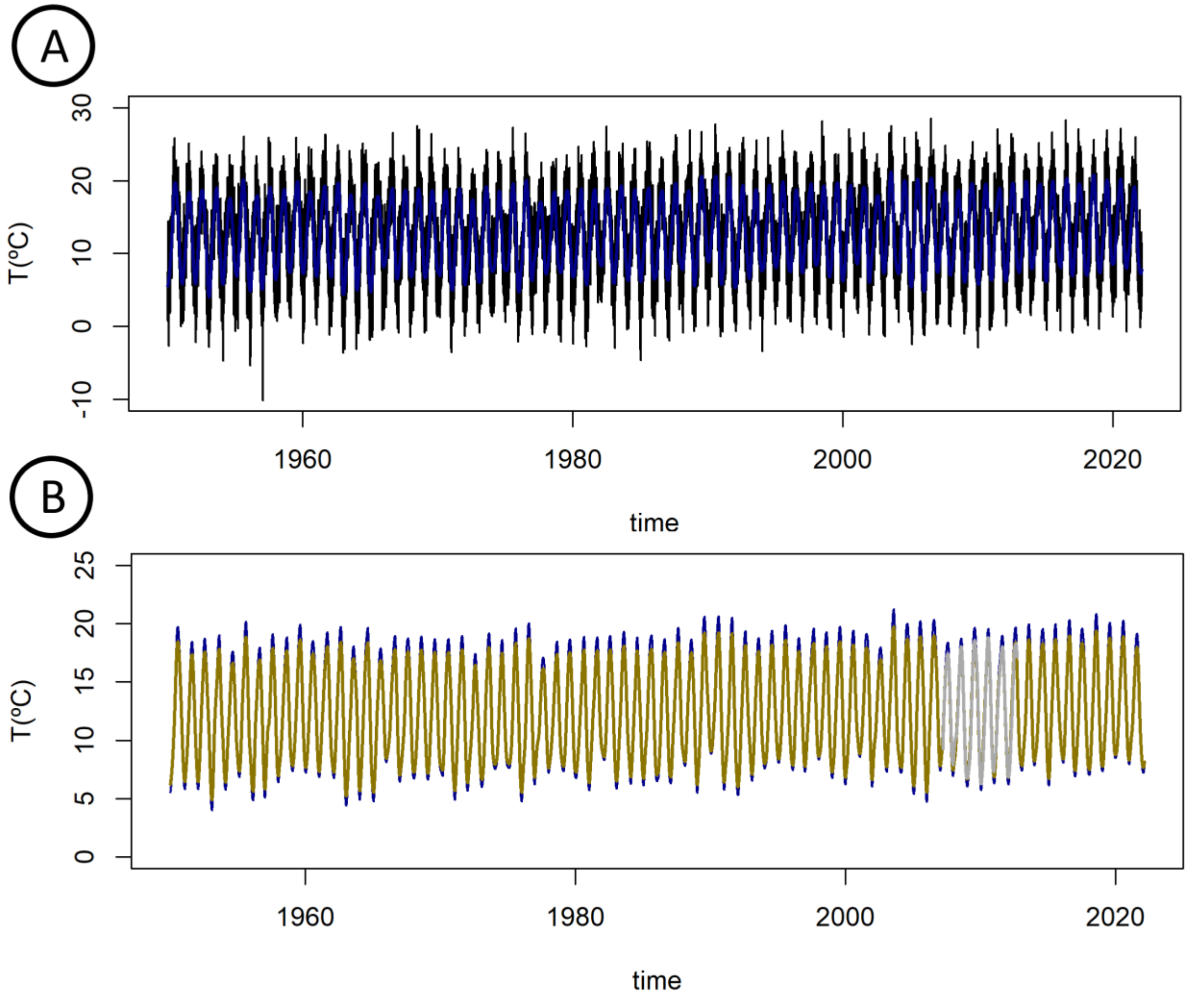

Fig. 9 Time series of temperature from the ECMWF model. Original product of the ERA5 simulations of the ECMWF model, before and after smoothing (A); time series before and after calibration (in dark blue and brown, respectively) with the *in situ* observations also reported in grey (B). The calibration parameters are provided in Supplementary Table 3.

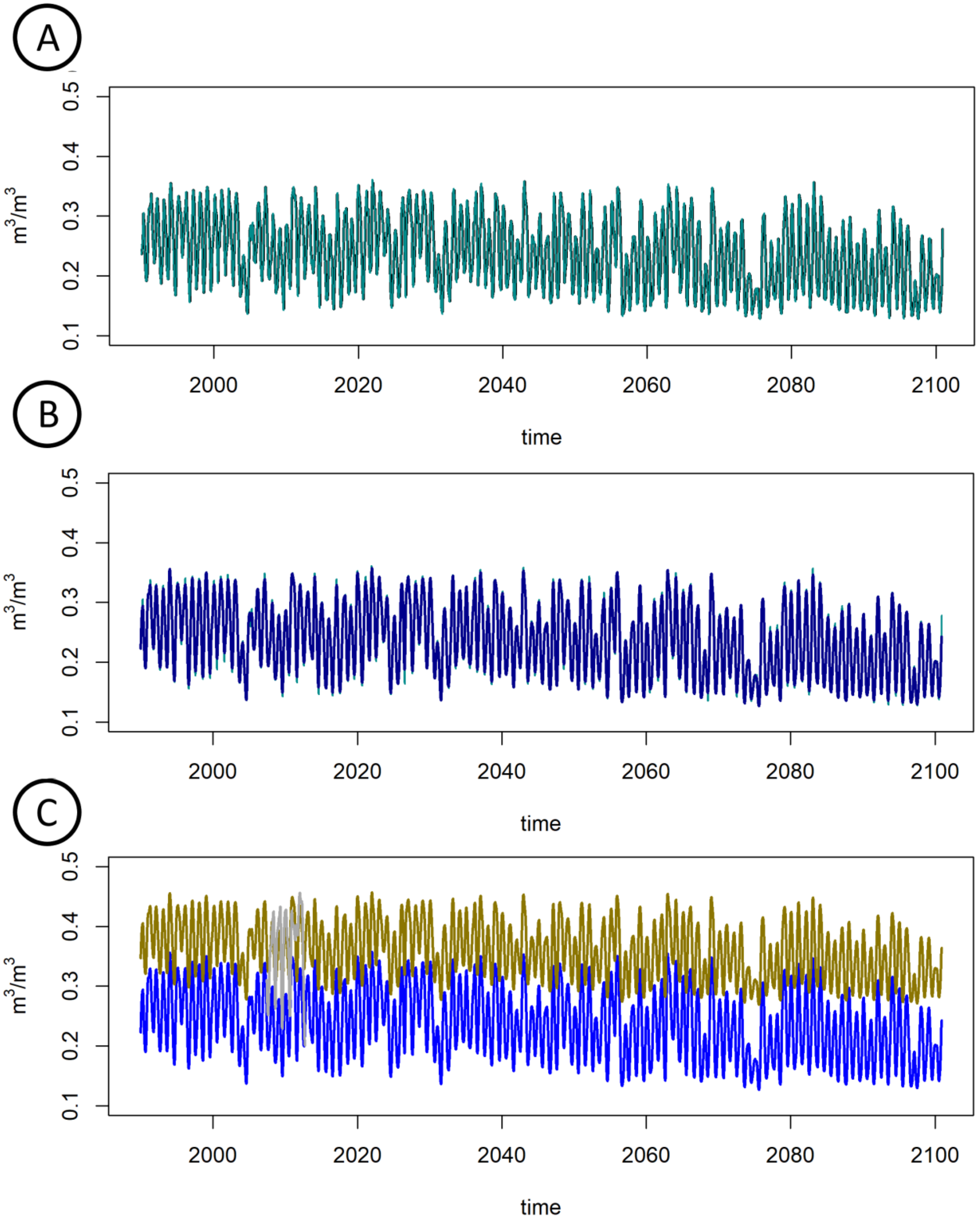

Fig. 10 Time series of volumetric water content from the IPCC A2 model. Original product of the IPCC A2 model, before and after resampling (A); smoothed time series before calibration (B), time series before and after calibration in blue and brown, respectively, with the *in situ* observations also reported in grey (C). The calibration parameters are provided in Supplementary Table 2.

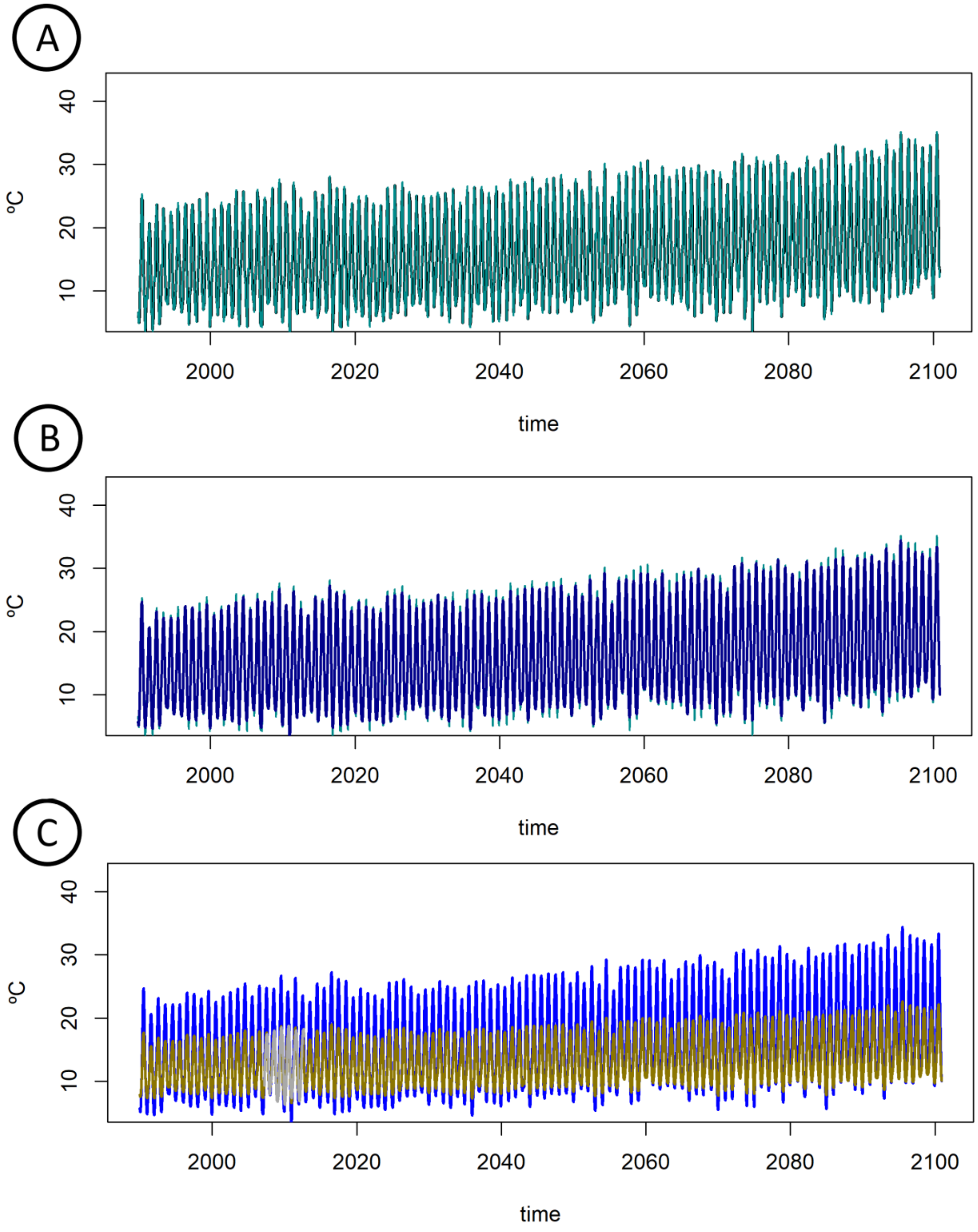

Fig. 11 Time series of temperature from the IPCC A2 model. Original product of the IPCC A2 model, before and after resampling (A); smoothed time series before calibration (B), time series before and after calibration in blue and brown, respectively, with the *in situ* observations also reported in grey (C). The calibration parameters are provided in Supplementary Table 3.

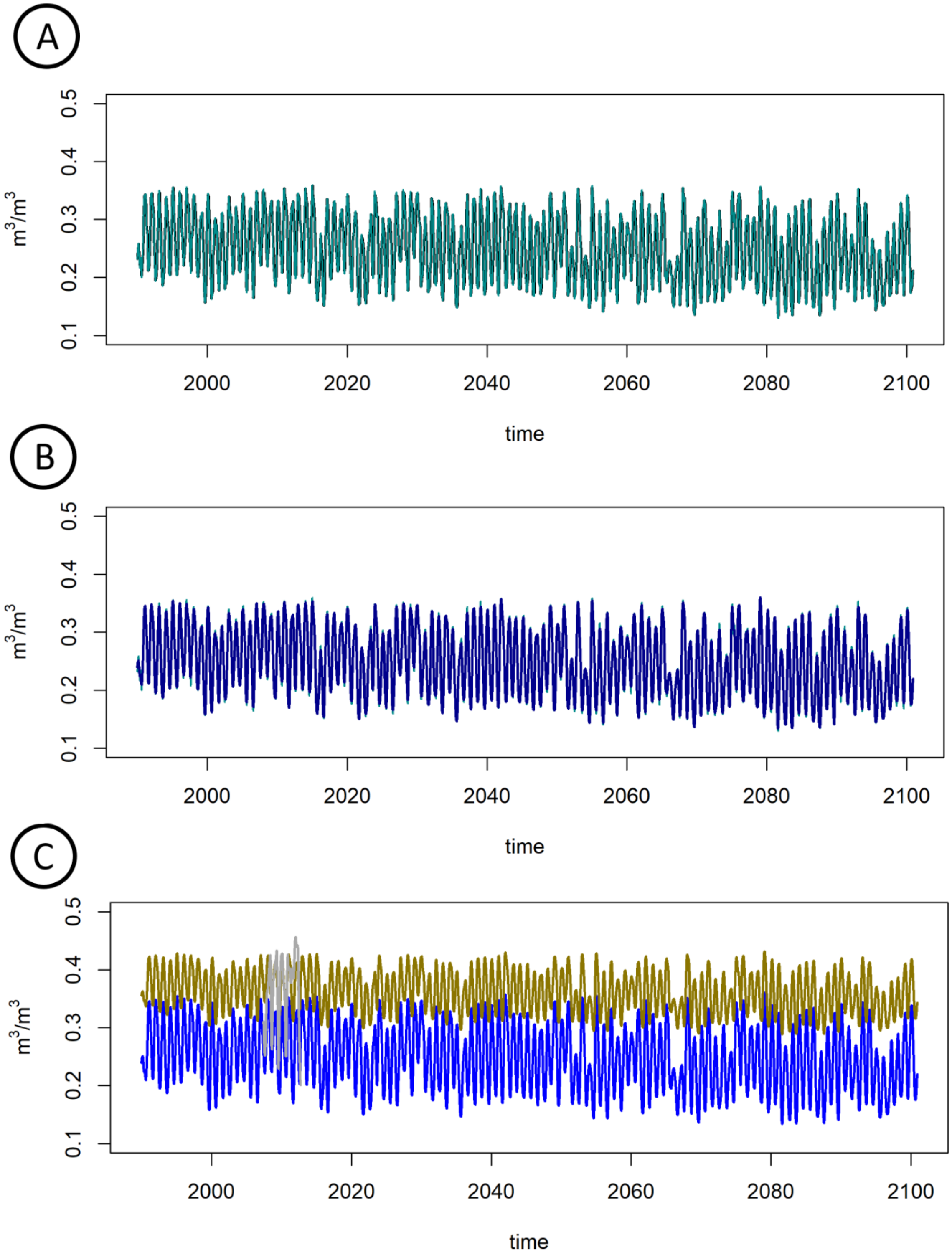

Fig. 12 Time series of volumetric water content from the IPCC B2 model. Original product of the IPCC B2 model, before and after resampling (A); smoothed time series before calibration (B), time series before and after calibration in blue and brown, respectively, with the *in situ* observations also reported in grey (C). The calibration parameters are provided in Supplementary Table 2.

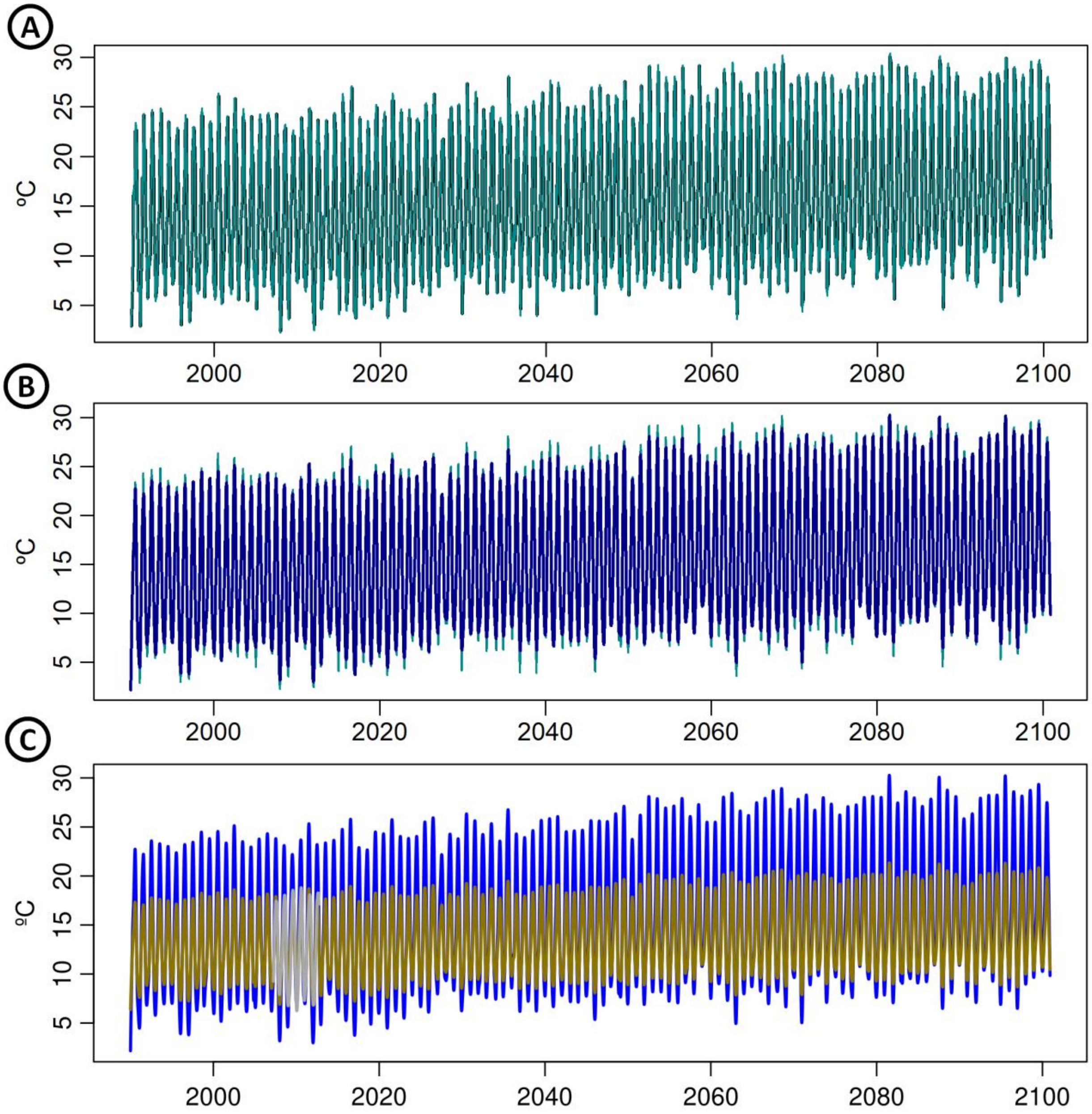

Fig. 13 Time series of temperature from the IPCC B2 model. Original product of the IPCC B2 model, before and after resampling (A); smoothed time series before calibration (B), time series before and after calibration in blue and brown, respectively, with the *in situ* observations also reported in grey (C). The calibration parameters are provided in Supplementary Table 3.

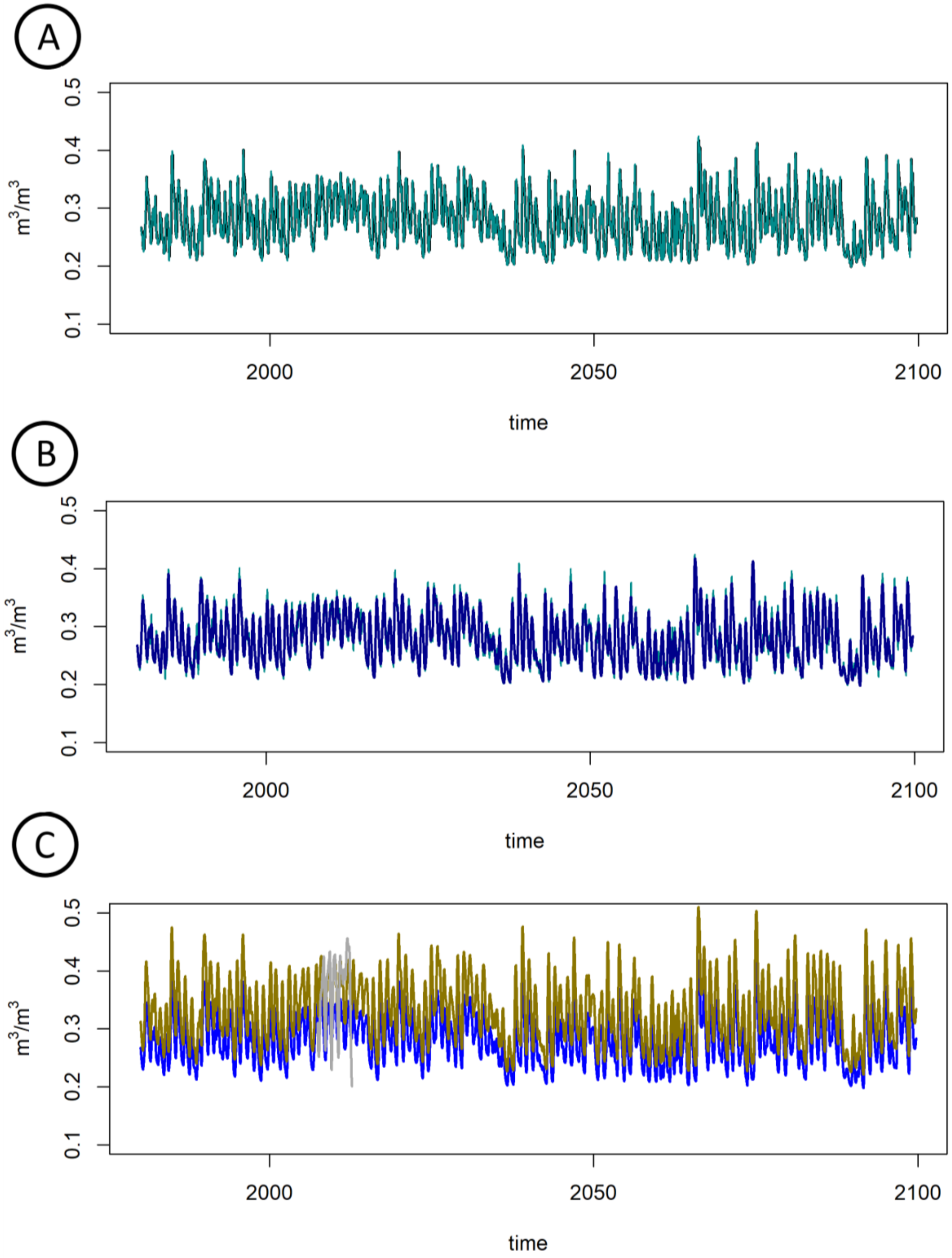

Fig. 14 Time series of volumetric water content from the IPCC A1B model. Original product of the IPCC A1B model, before and after resampling (A); smoothed time series before calibration (B), time series before and after calibration in blue and brown, respectively, with the *in situ* observations also reported in grey (C). The calibration parameters are provided in Supplementary Table 2.

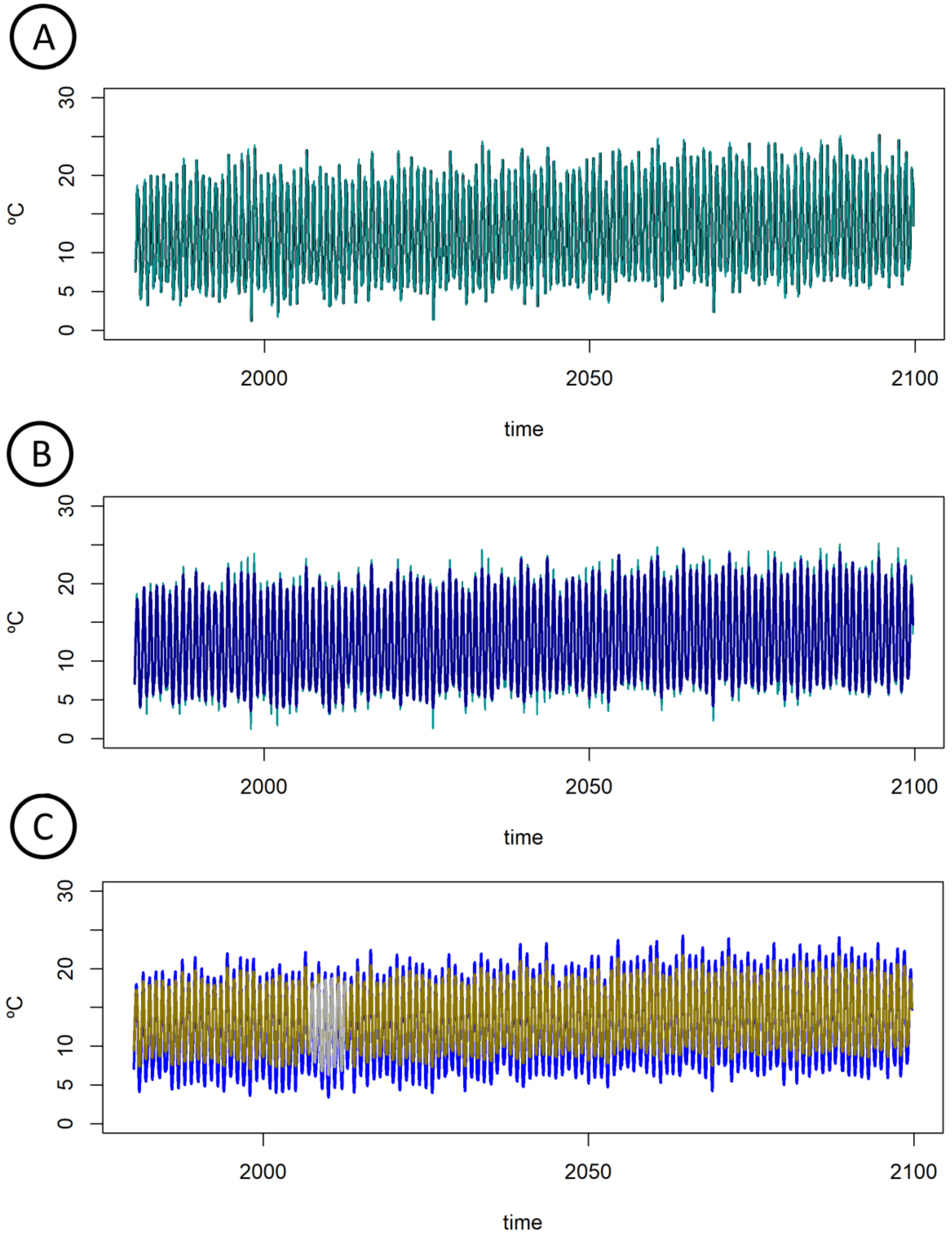

Fig. 15 Time series of temperature from the IPCC A1B model. Original product of the IPCC A1B model, before and after resampling (A); smoothed time series before calibration (B), time series before and after calibration in blue and brown, respectively, with the *in situ* observations also reported in grey (C). The calibration parameters are provided in Supplementary Table 3.

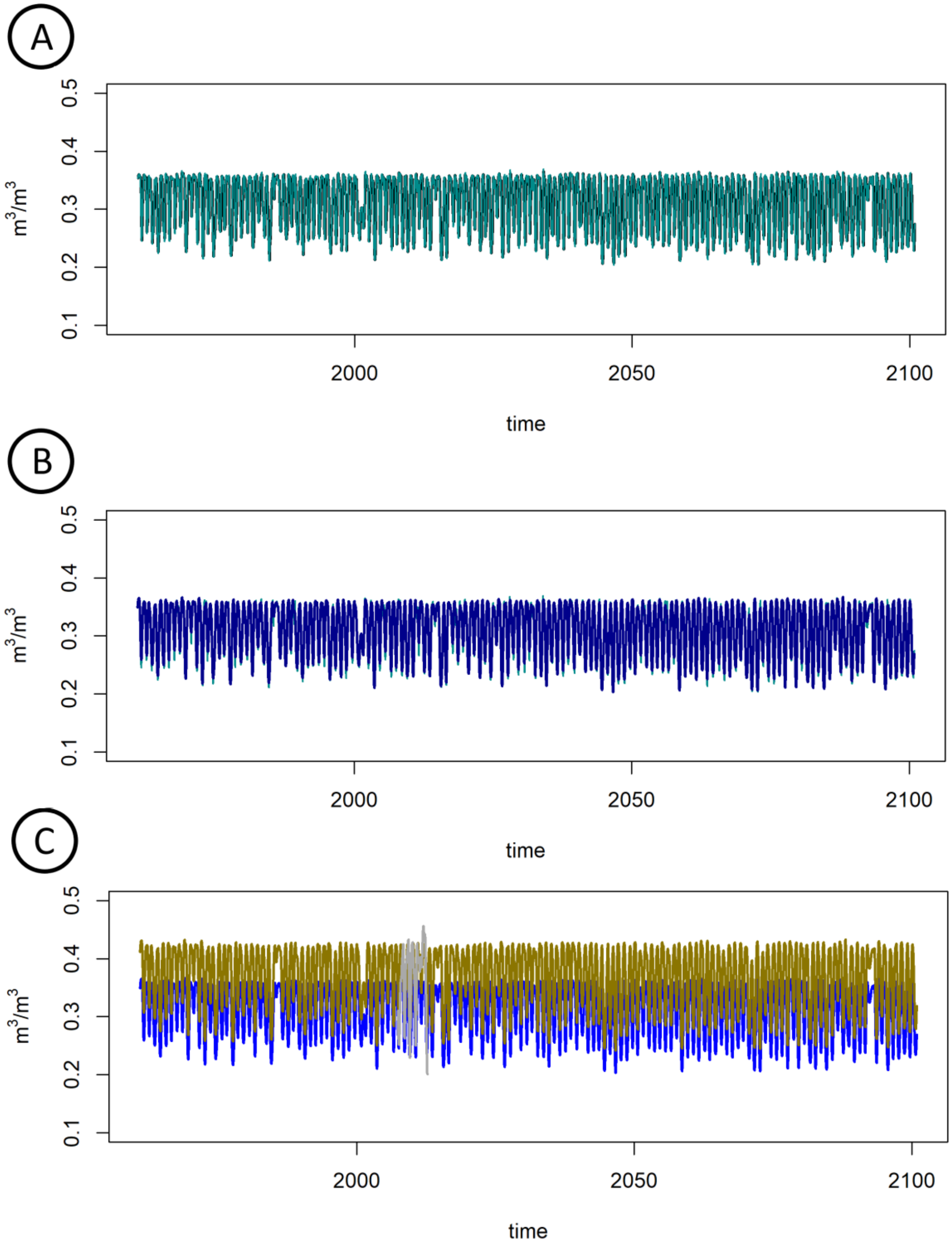

Fig. 16 Time series of volumetric water content from the IPCC B1 model. Original product of the IPCC B1 model, before and after resampling (A); smoothed time series before calibration (B), time series before and after calibration in blue and brown, respectively, with the *in situ* observations also reported in grey (C). The calibration parameters are provided in Supplementary Table 2.

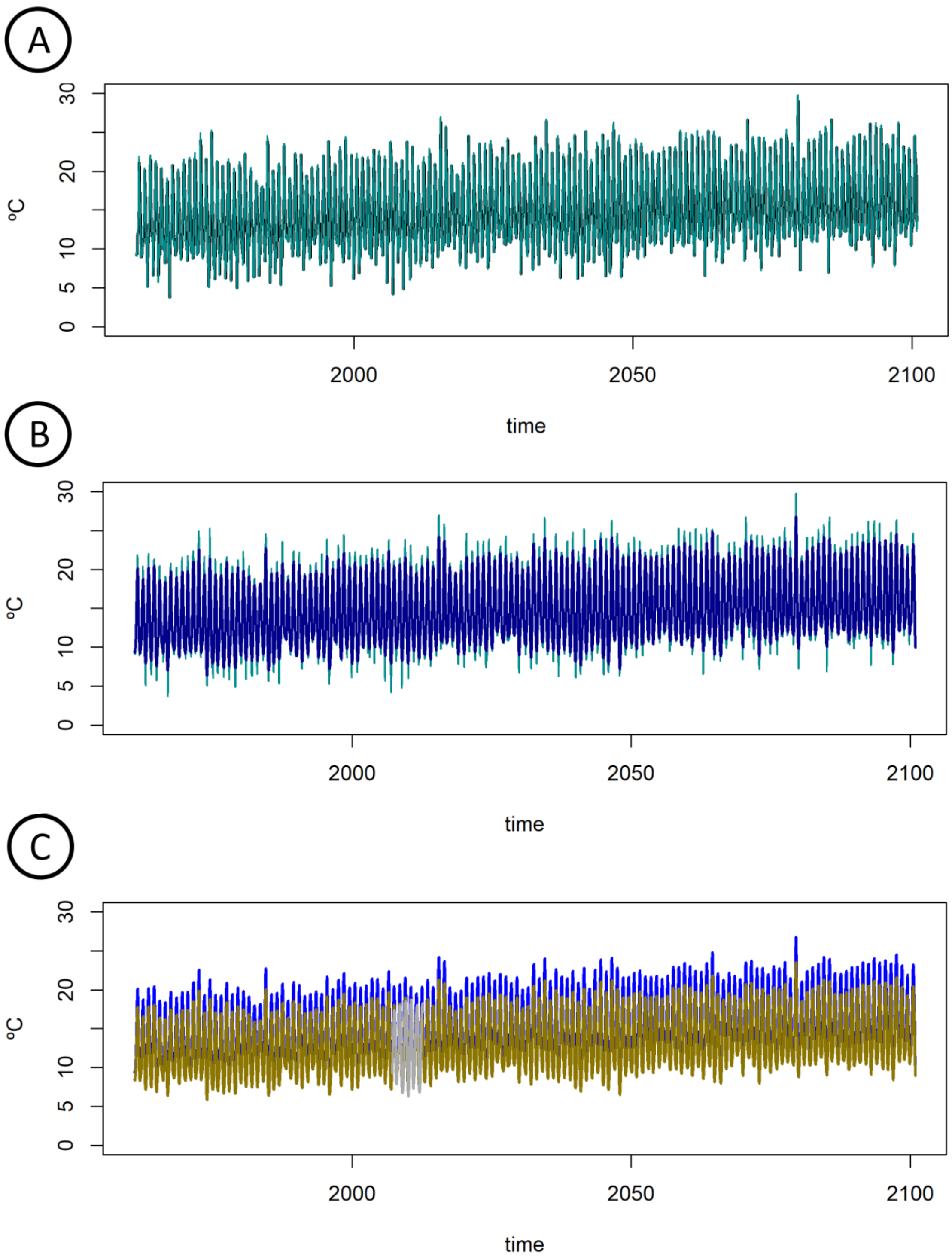

Fig. 17 Time series of temperature from the IPCC B1 model. Original product of the IPCC B1 model, before and after resampling (A); smoothed time series before calibration (B), time series before and after calibration in blue and brown, respectively, with the *in situ* observations also reported in grey (C). The calibration parameters are provided in Supplementary Table 3.

Table 1

Data sets considered in the study.

Obs.	Data set	Data type			Period	Resolution		Ref.
		In situ	satellite	hybrid		Time	Space	
				Volumetric Water Content (m^3/m^3)				
$W_{in\ situ}$	Altamira	×			2007-2012	≤ hourly*	-	*(72)*
W_{SMOS}	SMOS		×		2010-2020	2-3 days	15 km	*(75)*
W_{CCI}	ESA-CCI		×		1999-2020	≥ Daily*	0.25°	*(52)*
W_{ESM}	ESM			×	1970-2016	Monthly	0.5°	*(51)*
W_{IPCC}	ECHAM4 A1B			×	1990-2100	Monthly	2.8°	*(80)*
W_{IPCC}	ECHAM4 B1			×	1990-2100	Monthly	5.6°	*(81)*
W_{IPCC}	ECHAM4 A2			×	1990-2100	Monthly	2.8°	*(82)*
W_{IPCC}	ECHAM4 B2			×	1990-2100	Monthly	2.8°	*(83)*
				Temperature (°C)				
$T_{in\ situ}$	Altamira	×			2007-2012	≤ hourly*	-	*(72)*
T_{MODIS}	MODIS11 C3		×		2000-2021	Monthly	0.05°	*(53)*
T_{ECMWF}	ERA5			×	1950-2020	Hourly	0.1°	*(54)*
T_{IPCC}	ECHAM4 A1B			×	1990-2100	Monthly	2.8°	*(80)*
T_{IPCC}	ECHAM4 B1			×	1990-2100	Monthly	5.6°	*(84)*
T_{IPCC}	ECHAM4 A2			×	1990-2100	Monthly	2.8°	*(85)*
T_{IPCC}	ECHAM4 B2			×	1990-2100	Monthly	2.8°	*(86)*
				CO_2 concentration (ppm)				
$C_{in\ situ}$	Altamira	×			1997-1998	≤ hourly*	-	*(19)*
$C_{in\ situ}$	Altamira	×			2004-2005	≤ hourly*	-	*(15)*
$C_{in\ situ}$	Altamira	×			2007-2012	≤ hourly*	-	*(72)*
				Visitors (per year)				
V	Altamira	×			1952-2020	Yearly	-	*(Suppl. Table 11)*

* Full details are provided in the text (see Supplementary Note 2).

Table 2

Soil moisture time series inter-calibration.

Data set	Reference	Inter-calibration period	Calibration parameters		
			a(intercept)	*b*	R^2
ESM	*In situ*	2007-2012	0.046978 ± 0.004598	1.287757 ± 0.018322	56%
CCI	ESM	2005-2016	0.140969 ± 0.000837	1.018350 ± 0.003640	89%
ERA5	*In situ*	2007-2012	0.113178 ± 0.005350	0.819229 ± 0.017003	55%
ECHAM4 A1B	*In situ*	2007-2012	-0.04035 ± 0.01254	1.31905 ± 0.04047	36%
ECHAM4 B1	*In situ*	2007-2012	0.004263 ± 0.005474	1.167792 ± 0.017453	70%
ECHAM4 A2	*In situ*	2007-2012	0.165901 ± 0.004945	0.813436 ± 0.019586	47%
ECHAM4 B2	ESM	1990-2016	0.203945 ± 0.001071	0.632385 ± 0.003946	57%

Table 3

Temperature time series inter-calibration.

Data set	Reference	Inter-calibration period	Calibration parameters		
			a (intercept)	*b*	R^2
MODIS	*In situ*	2007-2012	0.245263 ± 0.071730	0.684060 ± 0.003769	89%
ERA5	*In situ*	2007-2012	1.430343 ± 0.033916	0.862765 ± 0.002448	98%
ECHAM4 A1B	*In situ*	2007-2012	4.42942 ± 0.06285	0.71445 ± 0.00493	91%
ECHAM4 B1	*In situ*	2007-2012	0.261956 ± 0.137883	0.869659 ± 0.009254	81%
ECHAM4 A2	*In situ*	2007-2012	4.879862 ± 0.116155	0.517352 ± 0.007042	73%
ECHAM4 B2	*In situ*	2007-2012	-140.237631 ± 1.941854	0.532518 ± 0.006759	76%

Table 4

Parameters tested in the modelling process and characteristics of the obtained models.

Models	$\mathbf{U}_C$	$\mathbf{U}_T$	$\mathbf{U}_W$	$\mathbf{M}_{TW}$	$\mathbf{M}_{CTW0}$	$\mathbf{M}_{CTW}$	$\mathbf{M}_C^*$
**Preprocessing**							
Data set	$C_{\text{in situ}}$	T_{MODIS}	W_{SMOS}	T_{MODIS},W_{SMOS}	C_{insitu},T_{insitu},W_{insitu}	$\mathbf{M}_{CTW0}$, $\mathbf{M}_{TW}$, $\mathbf{U}_T$	$\mathbf{M}_{CWT}$
Modelling period	2007-2009	2001-2006	2012-2019	2012-2019	2007-2009	-	-
Timeseries length (months)	27	66	79	79	28	-	-
Original data points number	78912	66	2255	2255	81792	-	-
# Re-sampled data points	2502	401	479	479	852	-	-
**GPoM**							
Modelling time step `tin` (hr)	8	120	120	120	24	-	-
S-Gfilt window length[×] `winL` (total points)	[9-25]	[9-21]	[9-21]	[9-21]	[9-21]	-	-
Model dimension `nVar` (n)	[3-5]	[3-4]	[3-4]	[3-5]	[3-5]	-	-
Max.pol. degr `dMax` (q^{max})	[2-5]	[2-5]	[2-5]	[2-3]	[2-3]	-	-
Constrained structure	No	No	No	Yes / No[××]	Yes / No[××]	-	-
**Postprocessing**							
Integr. method	RK4	RK4	RK4	RK4	RK4	RK4	RK4
Integration time step (day)	0.33	5	5	5	1 (0.5[×××])		1 (0.5[×××])
Integration time length(yr.)	300	300	1000	100	-	100	-
**Validation**							
Data set	$C_{\text{in situ}}$	T_{MODIS}	W_{SMOS}	T_{MODIS}, W_{SMOS}	C_{insitu},T_{insitu},W_{insitu}	C_{insitu},T_{insitu},W_{insitu}	C_{insitu}
Period	2009-2012	2000-2001, 2006-2021	2010-2012, 2019-2020	2010-2012, 2019-2020	2009-2012	2009-2012	2009-2012
H_P (model)(day)	30	30	30	30	30	30	30
H_P (indep.) (day)	30	30	30	30	30	30	30
**Characterization**							
Determinism detected	Yes	Yes	Yes	Yes	Yes	Yes	-
Lyapunov exp.: λ_1	$+8.8\cdot10^{-5}$ $\pm1.2\cdot10^{-5}$	$+9.7\cdot10^{-4}$ $\pm2.2\cdot10^{-4}$	$+1.4\cdot10^{-3}$ $\pm1.0\cdot10^{-4}$	-	-	-	-
λ_2	$-0.1\cdot10^{-5}$ $\pm1.5\cdot10^{-5}$	$+0.9\cdot10^{-4}$ $\pm1.3\cdot10^{-4}$	$-3.2\cdot10^{-4}$ $\pm2.6\cdot10^{-4}$	-	-	-	-
λ_3	$-9.7\cdot10^{-4}$ $\pm2.3\cdot10^{-5}$	$-7.7\cdot10^{-3}$ $\pm2.1\cdot10^{-4}$	$-3.5\cdot10^{-3}$ $\pm4.3\cdot10^{-5}$	-	-	-	-
D_{KY}	2.09 ±0.02	2.13 ±0.03	2.39 ±0.02	-	-	-	-
Folding detected	Yes	Yes	Yes	-	-	Yes	-

[×] Multiple options were tried since this parameter can determine whether models can be found or not. [××] Both unconstrainted and constrained algebraic structures were tried for the multivariate model (see methods section in the main text for further details). [×××] Due to the Runge-Kutta (RK4) method used to integrate the equations, a refined sampling time of the forcing time series was required in comparison to the other variables.

Table 5

Coefficients of polynomial P_C (Eq. S1 in Supplementary Note 3) for model $\mathbf{U}_C$.

Coefficient	Value
α_0	$-7.58029606246303 \cdot 10^{-7}$
α_1	$-3.12938229970615 \cdot 10^{-7}$
α_2	$+5.59475504129829 \cdot 10^{-9}$
α_3	$+2.44896870463009 \cdot 10^{-8}$
α_4	$+6.96377514531087 \cdot 10^{-10}$
α_5	$-5.13240541836387 \cdot 10^{-12}$
α_6	$-5.98297152320256 \cdot 10^{-11}$
α_7	$-6.15017855269830 \cdot 10^{-14}$
α_8	$+1.41498335799117 \cdot 10^{-15}$
α_9	$-2.09996296576249 \cdot 10^{-17}$
α_{10}	$-1.26862721810923 \cdot 10^{-19}$

Table 6

Coefficients of polynomial P_T (Eq. S2 in Supplementary Note 3) for model $\mathbf{U}_{T}$.

Coefficient	Value
μ_0	$8.20006395081456 \cdot 10^{-1}$
μ_1	$9.70076253639336 \cdot 10^{+2}$
μ_2	$4.51774044692980 \cdot 10^{+5}$
μ_3	$1.03060166273844 \cdot 10^{+8}$
μ_4	$1.14092469574834 \cdot 10^{+10}$
μ_5	$4.81439153312540 \cdot 10^{+11}$
μ_6	$3.18685528616669 \cdot 10^{-1}$
μ_7	$3.01762108828485 \cdot 10^{+2}$
μ_8	$1.05446578751559 \cdot 10^{+5}$
μ_9	$1.60463345444216 \cdot 10^{+7}$
μ_{10}	$8.89192686286158 \cdot 10^{+8}$
μ_{11}	$1.14065583127531 \cdot 10^{-3}$
μ_{12}	$2.97480278864674 \cdot 10^{-1}$
μ_{13}	$4.95073222494016 \cdot 10^{-2}$
μ_{14}	$3.51770055096173 \cdot 10^{+1}$
μ_{15}	$8.19872394620182 \cdot 10^{+3}$
μ_{16}	$6.24273686615096 \cdot 10^{+5}$
μ_{17}	$2.40677594285943 \cdot 10^{-4}$
μ_{18}	$4.21608125406015 \cdot 10^{-2}$
μ_{19}	$3.84282873034616 \cdot 10^{-3}$
μ_{20}	1.82125595792734
μ_{21}	$2.12351085836480 \cdot 10^{+2}$
μ_{22}	$1.72572520751691 \cdot 10^{-5}$
μ_{23}	$1.46415674369994 \cdot 10^{-3}$
μ_{24}	$1.49041846581378 \cdot 10^{-4}$
μ_{25}	$3.53352160341646 \cdot 10^{-2}$
μ_{26}	$4.12062545914954 \cdot 10^{-7}$
μ_{27}	$2.31061932756596 \cdot 10^{-6}$

Table 7

Coefficients of polynomial P_W (Eq. S3 in Supplementary Note 3) for model $\mathbf{U}_W$.

Coefficient	Value
τ_0	$+4.97777271966761\cdot10^{-5}$
τ_1	–0.53565938321849
τ_2	+952.807782485174
τ_3	+ 0.00259744303401131
τ_4	–211.14170107848
τ_5	+ 1007184.85266376
τ_6	–1.13132432667175
τ_7	+ 254.551793389073
τ_8	–0.000559193235819823
τ_9	+3.79850472696198
τ_{10}	–2376.93871305567
τ_{11}	–0.0327098645276611
τ_{12}	+1173.58658465871
τ_{13}	–2805535.73018167
τ_{14}	+6.4857439349921
τ_{15}	–940.153296624794
τ_{16}	+0.00233770454165381
τ_{17}	–8.84540652357964
τ_{18}	+0.112582081762738
τ_{19}	–1645.06721890306
τ_{20}	–9.90205846586652
τ_{21}	–0.00430204273073802
τ_{22}	+6.72856277688911
τ_{23}	–0.119166058634181
τ_{24}	+0.00293838966627193

Table 8

Forecasting errors (90% percentile of the relative error of CO_2 concentration, temperature and Volumetric Water Content) in the identification and validation windows for different horizons of prediction.

	CO_2 concentration		Temperature		VWC	
	C (ppm)		*T* (°C)		*W* (m^3/m^3)	
Horizon of prediction (day)	Modelling window	Validation window	Modelling window	Validation window	Modelling window	Validation window
5	0.023	0.032	0.202	0.211	0.331	0.368
10	0.182	0.298	0.407	0.423	0.678	0.741
15	0.625	0.968	0.615	0.627	1.031	1.046
20	1.485	2.102	0.827	0.849	1.403	1.330

Table 9

Model coefficients (including α_9) of model $\mathbf{M}_{CTW}$ (Eqs. 15-20).

Coefficient	Value
α_0	$2.4860083461707779 \cdot 10^{+2}$
α_1	$1.1131673948998334 \cdot 10^{+3}$
α_2	$1.2184817703275930 \cdot 10^{+3}$
α_3	$4.1781706465875857 \cdot 10^{+2}$
α_4	$2.1490965302182740 \cdot 10^{+1}$
α_5	$8.5975101206193187 \cdot 10^{+1}$
α_6	$7.2472289874368258 \cdot 10^{+1}$
α_7	$3.9890558062520964 \cdot 10^{-1}$
α_8	1.1618577320640777
α_9	$8.3235587095475665 \cdot 10^{-3}$
θ_1	$3.31926472022979 \cdot 10^{-3}$
θ_2	$6.17813737026041 \cdot 10^{-7}$
θ_3	$4.94961109184413 \cdot 10^{-4}$
θ_4	$4.53325932714220 \cdot 10^{-8}$
θ_5	$1.73421600002026 \cdot 10^{-5}$
γ_0	$2.28015736842223 \cdot 10^{-3}$
γ_1	$1.62224759041476 \cdot 10^{-2}$
γ_2	$2.34444650815511 \cdot 10^{-2}$
γ_3	$2.63510498510386 \cdot 10^{-1}$
γ_4	$6.23933979722772 \cdot 10^{-1}$
γ_5	$1.87426100619980 \cdot 10^{-4}$
γ_6	$2.62950498274644 \cdot 10^{-4}$
γ_7	$9.45569894881387 \cdot 10^{-4}$
γ_8	$2.50786782221198 \cdot 10^{-5}$
γ_9	$2.62610976760350 \cdot 10^{-5}$
γ_{10}	$4.86534645923887 \cdot 10^{-7}$

Table 10

Forecasting errors (90% percentile of the relative error of CO_2 concentration in ppm) in the identification and validation windows for different horizons of prediction.

	CO_2 concentration C (ppm)			
	Not real forcings		Real forcings	
Horizon of prediction (day)	Modelling window	Validation window	Modelling window	Validation window
5	2.907	3.912	3.455	3.763
10	5.275	6.551	6.328	7.670
15	7.294	9.318	8.568	10.590
20	9.828	10.165	11.519	12.460

Table 11
Number of visitors per year in the cave (information provided by the Museum of Altamira).

Year	Visitors
1952	30000
1953	33000
1954	37000
1955	46000
1956	52000
1957	62000
1958	57000
1959	60886
1960	59963
1961	67000
1962	72000
1963	90000
1964	110000
1965	118000
1966	127000
1967	155000
1968	152000
1969	155000
1970	157000
1971	155731
1972	174613
1973	174296
1974	162072
1975	151326
1976	83643
1977	36693
1982	8000
1983	11320
1984	11320
1985	11320
1986-2001	11320
2002	5860
2014	272
2015-2019	336
2020	194
2021-2022	336

Supplementary material (in a separate file)

In this data file, the time series used to run the scenarios are provided. These include the time series of volumetric water content from SMOS (2010-2020), ESA-CCI (1999-2020), ESM (1970-2016) and ECHAM4 (1990-2100), and of temperature from LST (2000-2021), ERA5 (1950-2020) and ECHAM4 (1990-2100). For the *in situ* time series, please contact with the authors.
